# Regulation of Juvenile Hormone on Summer Diapause of *Geleruca daurica* and Its Pathway Analysis

**DOI:** 10.3390/insects12030237

**Published:** 2021-03-11

**Authors:** Hong-Yue Ma, Yan-Yan Li, Ling Li, Yao Tan, Bao-Ping Pang

**Affiliations:** Research Center for Grassland Entomology, Inner Mongolia Agricultural University, Hohhot 010020, China; plmmhy1111@163.com (H.-Y.M.); lyy4455@163.com (Y.-Y.L.); lling@imau.edu.cn (L.L.); 850310.tanhuaf4@163.com (Y.T.)

**Keywords:** *Galeruca daurica*, juvenile hormone, methoprene, diapause, RNA-Seq

## Abstract

**Simple Summary:**

Diapause is an arrestment state in development, and plays an important role in life history in insects. It has been thought that a lack in juvenile hormone (JH) results in reproductive diapause occurring at the adult stage. However, we do not fully know about the underlying molecular mechanism. In this study, we proved that the topical application of a JH analog methoprene caused the changes at the transcriptional levels of a great number of genes, inhibited lipid accumulation, and finally delayed the adults entering diapause. Therefore, JH signaling plays an important role in regulating reproductive diapause of *G. daurica*, a new pest with great outbreaks in Inner Mongolia.

**Abstract:**

Juvenile hormone (JH) signaling plays an important role in regulation of reproductive diapause in insects. However, we have little understanding of the effect of JH on gene expression at the transcriptome level in diapause. *Galeruca daurica* is a new pest in the Inner Mongolia grasslands with obligatory summer diapause in the adult stage. Topical application of a JH analog methoprene at the pre-diapause stage delayed the adults entering diapause and inhibited lipid accumulation whereas it did not during diapause. Using Illumina sequencing technology and bioinformatics tools, 54 and 138 differentially expressed genes (DEGs) were detected at 1 and 2 d after treatment, respectively. The KEGG analysis showed that the DEGs were mainly enriched in the metabolism pathways. qRT-PCR analysis indicated that methoprene promoted the expression of genes encoding vitellogenin, fork head transcription factor and Krüppel homolog 1, whereas suppressed the expression of genes encoding juvenile hormone-binding protein, juvenile hormone esterase, juvenile hormone acid methyltransferase, juvenile hormone epoxide hydrolase and fatty acid synthase 2. These results indicate that JH signaling plays an important role in regulating reproductive diapause of *G. daurica*.

## 1. Introduction

Diapause is an adaptive strategy of insects to overcome adverse environmental seasons and ensure population continuity [1]. Diapause can occur at any developmental stage, such as embryonic, larval, pupal and adult stages, however, for any given species, diapause usually occurs at a single stage [2]. Adult diapause is also known as reproductive diapause, and its central feature is the suppression of reproductive functions such as maturing of ovaries and male accessory glands, and mating activity [3]. Adult diapause can be usually attributed to the absence of juvenile hormone (JH) [4]. JH is a family of sesquiterpenoids, and is known to play a critical role in regulating insect metamorphosis, reproduction, and diapause [5,6]. The molecular basis of the JH signaling pathway has been recently unveiled [7,8,9,10,11]. Many studies have suggested that exogenous JH analogue (JHA) would mimic the action of JH and likely act directly on the JH receptors as a substitute for JH [12]. JHAs have been effectively used to understand the mechanism of JH action. Topical applications of JH and several analogues one day after the initiation of diapause could break diapause 8–10 days later, whereas JH application during pre-diapause only delayed diapause in *Leptinotarsa decemlineata* [13]. Since then, many studies have reported that the application of exogenous JH or JHA can terminate adult diapause and induce ovarian development or oviposition in a number of beetle species [14]. The exposure to JHA methoprene promoted the development of ovaries and terminated reproductive diapause in *Melinda pusilla* [15] and *Polygonia c-aureum* [16]. Lipids provide an important energy reserve for diapausing insects, and understanding the molecular mechanisms involved in lipid accumulation during diapause preparation is indispensable to understand the metabolic processes regulating diapause in insects [17]. FASs are highly conserved and crucial genes in the fatty acid biosynthetic pathway [18]. JHA treatment repressed the expression of *FAS2* in *C. bowringi*, and knockdown of *FAS2* by RNAi suppressed lipid accumulation [19]. However, up to now, the above studies have focused on facultative diapause, and the molecular basis of this hormonal regulation on reproductive diapause has not been fully understood [20]. To the best of our knowledge, it has been not reported how JH or JHA influences the gene expression in reproductive diapause at a transcriptome level.

*Galeruca daurica* (Joannis) (Coleoptera—Chrysomelidae) has become one of the most destructive pests on the grasslands of Inner Mongolia since its sudden outbreak in 2009, and has caused severe economic losses and grassland degradation [21]. This pest is an univoltine species with obligatory summer diapause in the adult stage. Overwintering eggs start to hatch in mid-April. From late May to early June, after newly-emerged adults feed for about one week, the beetles begin to aggregate under grass, cow dung and stone, and enter diapause. About three months later, adults terminate diapause and reactivate, and lay eggs in autumn [22]. Our previous studies suggested that the JH signaling pathway might play a crucial role in regulating diapause in *G. daurica* [23,24]. In this study, in order to clarify the effect of JH on obligatory reproductive diapause and its global gene regulatory cascades, we used the next-generation high-throughput sequencing technology (RNA-Seq) to analyze the transcriptional changes in response to topical JHA treatment in the *G. daurica* adults, measured the effects of JHA on the total lipid content and diapause rate of *G. daurica*, and finally compared the expression levels of the eight JH-related genes after JHA application by qRT-PCR. The results revealed a great number of JH target genes and metabolic pathways. Therefore, our study provides a solid foundation for further elucidation of the regulatory cascades in JH-controlled reproductive diapause.

## 2. Materials and Methods

### 2.1. Insect Rearing

Eggs were originally collected from the Xilingguole grasslands of Inner Mongolia, China, on 15 April 2019. The insects were reared in the Research Center for Grassland Entomology, Inner Mongolia Agricultural University. Eggs were incubated in an incubator (temperature: 25 ± 1 °C; RH: 80 ± 5%; photoperiod: 14 h light: 10 h dark). Insects were reared individually in Petri dishes (9.0 cm in diameter and 1.5 cm in height) lined with moist filter papers under natural conditions after larval emergence. *Allium mongolium* was provided as food once a day.

### 2.2. JHA Treatment

JHA methoprene (CAS-number: 40596-69-8; purity: 98.0%; MedChemExpress, China) was diluted to 25 mg/mL concentrations using dimethyl sulfoxide (DMSO) and stored at −80 °C as a stock solution, and then diluted to 1.0 and 2.5 μg/μL working concentrations with normal saline. A DMSO solution diluted with normal saline was used as a negative control, and untreated adults as blank controls. Two microlitres of control solution or treatment liquor was injected into the adult abdomens using a microsyringe.

### 2.3. RNA Extraction and cDNA Synthesis

Total RNA was extracted using RNAiso plus reagent (TaKaRa. Dalian, China) according to the manufacturer’s instructions. RNA concentration and purity were determined using a NanoPhotometer^®^ P330 microspectrophotometer (Implen, Munich, Germany). The integrity of RNA was detected using electrophoresis in 1% agarose gel. The RNA was treated with DNAase to eliminate residual genomic DNA and then complementary DNA (cDNA) was synthesized for qPCR using the PrimeScript^®^ RT Reagent Kit with gDNA Eraser (TaKaRa, Dalian, China) and stored at −20 °C until use.

### 2.4. Transcriptome Sequencing, Assembly and Bioinformatics Analysis

For transcriptome analysis, two microlitres of methoprene (2.5 μg/μL) was topically applied to the adults at 3 days after eclosion. After JHA treatment, individuals were sampled at 1 (Ta) and 2 days (Tb) post-injection, respectively, and individuals treated with carrier solution served as control groups (CKa, 1 day post-injection; and CKb, 2 days post-injection). Three biological replicates of each treatment with at least 10 individuals (5 females and 5 males) per replication were performed. Samples were sent to Biomarker Technologies Co, LTD (Beijing, China) for transcriptome sequencing. The sequencing libraries were generated using NEBNext^®^Ultra^™^ RNA Library Prep Kit for Illumina^®^ (NEB, Ipswich, MA, USA) following manufacturer’s recommendations. To select cDNA fragments of preferentially 240 bp in length, the library fragments were purified with the AMPure XP system (Beckman Coulter, Beverly, MA, USA). Library quality was assessed on the Agilent Bioanalyzer 2100 system. The library preparations were sequenced on the Illumina Hiseq 6000 platform and paired-end reads were generated. Clean data were obtained by removing reads containing adapter, ploy-N and low-quality read from raw data. At the same time, Q20, Q30, GC-content, and sequence duplication levels of the clean data were calculated. De novo assembly of RNA-Seq was performed based on the left.fq and right.fq using Trinity [25] with min_kmer_cov set to 2 by default and all other parameters set default. The annotation of assembled sequences was performed by BLASTn and BLASTx (E-values ≤ 1 × 10^−5^) based on the following databases—Nr (NCBI on-redundant protein sequences), Nt (NCBI non-redundant nucleotide sequences), Pfam (Protein family), KOG/COG (Clusters of Orthologous Groups of proteins), Swiss-Prot (A manually annotated and reviewed protein sequence database), KO (KEGG Ortholog database) and GO (Gene Ontology).

Transcript expression level was estimated in combination with RSEM (RNA-Seq by Expectation-Maximization) [26]. The FPKM (Fragments Per Kilobase of transcript per Million mapped reads) value was used to represent the expression level of corresponding transcripts. Differential expression analysis was performed using the DESeq2 [27]. The Benjamini and Hochberg approaches were used to adjust *p* values (false discovery rate, FDR). Genes with *FDR* < 0.05 and *FC* (Fold change) ≥ 2 were assigned as differentially expressed genes (DEGs). Gene Ontology (GO) enrichment analysis of DEGs was performed by GOseq R packages based on Wallenius non-central hyper-geometric distribution [28]. KOBAS software was used to test the statistical enrichment pathways of DEGs in the Kyoto Encyclopedia of Genes and Genomes (KEGG) [29].

### 2.5. Quantitative Real-Time PCR

To analyze the expression of the JH-induced genes, the treated insects were collected at 1, 2, 4, 6, 8, 10, and 12 days after JHA application. Quantitative real-time PCR (qRT-PCR) was used to determine the expression levels of 8 genes related to the JH pathway after JHA treatment, including *JHBP* (juvenile hormone binding protein), *JHE* (juvenile hormone esterase), *JHAMT* (juvenile hormone acid methyltransferase), *JHEH* (juvenile hormone epoxide hydrolase), *Vg* (vitellogenin), *FOXO* (fork head transcription factor), *Kr-h1* (Krüppel homolog 1), and *FAS2* (fatty acid synthase 2). These 8 genes were selected based on our RNA-Seq database assembled in this study and documents [5,19]. The gene-specific primers were designed using an online qRT-PCR primer design tool (http://www.ncbi.nlm.nih.gov/tools/primer-blast/ (accessed on 15 September 2020)) based on our RNA-Seq data of *G. daurica* (Table S1). The qRT-PCR reaction was conducted using the BRYT Green^®^ dye kit (Promega, Fitchburg, WI, USA) and the FTC-3000P Real-Time Quantitative Thermal Cycler systems (Funglyn Biotech, Richmond Hill, ON, Canada). The succinate dehydrogenase complex (*SDHA*) was used as the reference gene [21]. qRT-PCR reactions were performed in 20 μL mixture, which consisted of 2 μL of cDNA, 0.4 μL of forward and reverse primer (10 μmol/L), 10 μL GoTaq^®^ qPCR Master Mix, and 7.2 μL of RNase-free H_2_O. All reactions used the following amplification conditions—initial denaturation at 95 °C for 10 min, followed by 40 cycles at 95 °C for 15 s, 60 °C for 1 min, and 72 °C for 15 s. The qRT-PCR data were analyzed by the 2 ^−∆∆Ct^ method [30] based on three biological replicates and four technical replicates. Each biological replicate included 3 females and 3 males.

### 2.6. Total Lipid Content Measurement

The total lipid content was determined according to the chloroform–methyl alcohol method [31]. Briefly, the treated insects were placed in a centrifuge tube, and dried in an oven at 60 °C for 3 days, weighed to measure dry mass (DM), then ground in 1 mL chloroform–methanol (2:1) solution and then centrifugation occurred. The lean dry mass (LDM) was measured after placed in an oven at 60 °C and baked to constant weight. The fat content (FC) was calculated by FC = (DM − DM)/LDM. Three independent biological replicates were performed for each treatment, and each replicate included 5 females and 5 males and one individual was tested each time. Therefore, the FC value of each replicate was calculated as a mean of 10 values (individuals), and the FC value of each treatment was calculated as a mean of three replicates (30 individuals).

### 2.7. Behavioral and Developmental Observation

To investigate the effects of JHA (methoprene) on the development of *G. daurica* adults, the topical applications of methoprene of two concentrations (2.5 and 1.0 μg/μL) were performed on the adults at 3, 5, and 15 days post-eclosion, respectively. Each treatment included three biological replicates and each replicate contained 25 males and 25 females. The beetles were observed twice a day for feeding behavior and we recorded the number of feeding adults every day after treatment. When adults began to stop feeding continuously, diapause entry was determined, because our previous observation indicated that adults started to stop feeding about one week after eclosion and enter diapause with sudden decreasing in respiration intensity [22,23].

### 2.8. Statistical Analysis

Differences in the expression levels of each target gene after JHA treatments were analyzed using one-way analysis of variance (ANOVA) followed by Tukey’s Honestly Significant Difference (HSD) tests (*p* < 0.05). All datasets were analyzed using SPSS 20.0 software. All data were presented as the mean ± SE (standard error).

## 3. Results

### 3.1. Effects of JHA on the Development of G. daurica Adults

**Figure 1:**
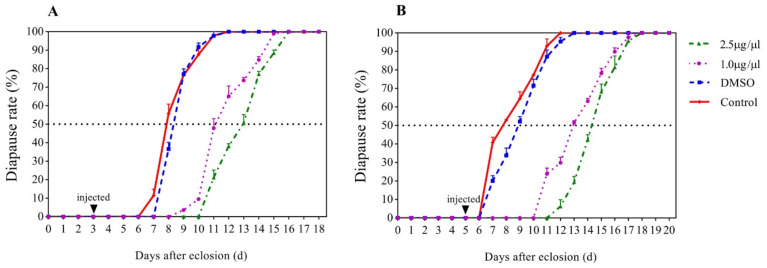
Effects of juvenile hormone analogue (JHA) JHA on diapause rates of *G. daurica* adults. (**A**) JHA application at 3 days post-eclosion. (**B**) JHA application at 5 days post-eclosion.

The results showed that JHA treatments at 3 and 5 days post-eclosion affected significantly the development of adults but the 15-day treatment did not (Figure 1). When JHA applications were conducted at 3 days post-eclosion, the diapause rates of JHA (2.5 μg/μL), JHA (1.0 μg/μL), solvent control, and blank control reached 50% at 12.90, 11.10, 8.34, and 7.84 days post-eclosion, respectively. With exposure to 2.5 and 1.0 μg/μL JHA, the diapause rates at 50% were delayed by 4.56, and 2.76 days, respectively, compared with the solvent controls (Figure 1A). When JHA applications were conducted at 5 days post-eclosion, the diapause rates of JHA (2.5 μg/μL), JHA (1.0 μg/μL), solvent control, and blank control reached to 50% at 14.30, 12.90, 8.88, and 7.72 days post-eclosion, respectively. With exposure to 2.5 and 1.0 μg/μL JHA, the diapause rates at 50% was delayed by 5.42 and 4.02 days, respectively, compared with the solvent control (Figure 1B). However, when JHA applications were conducted at 15 days post-eclosion, all treated individuals still kept diapause. The results indicated that JHA application significantly postponed diapause initiation when the treatment was performed at the pre-diapause stage (3 and 5 days after eclosion), whereas it did not affect diapause when the treatment was conducted during diapause (15 days after eclosion) in *G. daurica*.

### 3.2. Effects of JHA on the Total Lipid Content in G. daurica

**Figure 2:**
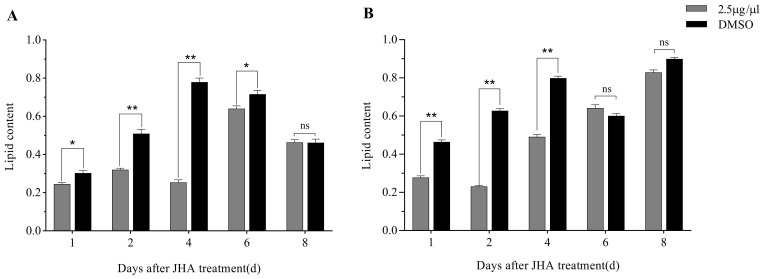
Effects of JHA on the total lipid content of *G. daurica* adults. (**A**) JHA application 3 days after adult eclosion. (**B**) JHA application 5 days after adult eclosion. The data are shown as the mean ± SE. * and ** indicate significant difference at *p* < 0.05 and *p* < 0.01, respectively, and ‘ns’ means no significant difference at *p* < 0.05 (Student’s *t*-test).

The effects of JHA treatments at 3 and 5 days post-eclosion on the total lipid content are shown in Figure 2. The result showed that JHA treatments affected significantly the lipid content in the *G. daurica* adults. When JHA was applied at 3 days post-eclosion, the lipid contents in JHA treatment were significantly less than those in the DMSO controls from 1 to 6 days after treatment (Figure 2A). When JHA was applied at 5 days post-eclosion, the lipid contents in JHA treatment were significantly less than those in the DMSO controls from 1 to 4 days after treatment (Figure 2B). These results showed that JHA application at the pre-diapause stage could inhibit the lipid accumulation in the *G. daurica* adults, and this inhibitory effect lasted for at least 4 days.

### 3.3. Transcriptome Changes in Response to JHA Application

**Figure 3:**
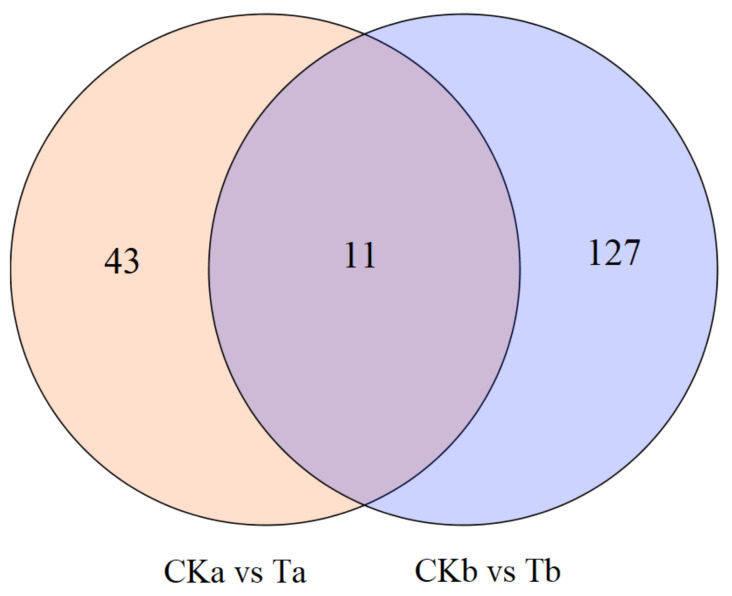
Venn diagram of differentially expressed genes after JHA application. CKa and CKb (controls)—1 and 2 days post-injection with carrier solution, respectively; Ta and Tb (treatments)—1 and 2 days post-injection with methoprene (2.5 μg/μL), respectively.

To reveal the molecular mechanism of JH signaling in diapause regulation at the transcriptional level in *G. daurica*, RNA-Seq was performed for the adults after JHA treatments. A total of 87,236 unigenes with an average length of 838.12 bp and an N50 of 1677 bp were obtained by Trinity assembly from 12 samples (CKa1, CKa2, CKa3, CKb1, CKb2, CKb3, Ta1, Ta2, Ta3, Tb1, Tb2, Tb3) (Table S2). Compared with the control (CKa), 54 DEGs (34 up-regulated and 20 down-regulated) were obtained one day after JHA application, and compared with the control (CKb), 138 DEGs (48 up-regulated and 90 down-regulated) were found at 2 days after JHA application. Among them, 11 common DEGs (four up-regulated and seven down-regulated) were identified (Figure 3 and Table 1). Finally, all of the DEGs were characterized by BLAST to investigate the homologous protein-coding genes annotated in the Nr database (Table S3).

**Figure 4:**
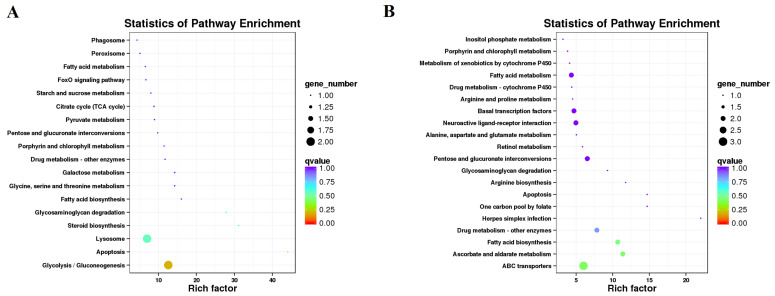
Enriched KEGG pathways for differentially expressed genes after JHA application. (**A**) One day after JHA application. (**B**) Two days after JHA application.

To analyze the putative molecular mechanisms of JHA effects on reproductive diapause, the annotated DEGs were subjected to the KEGG and GO enrichment analysis. For the CKa vs. Ta comparison, eight DEGs were enriched in the 18 KEGG pathways, among which nine pathways (50%) belonged to metabolism pathways, and the main enrichment pathways included apoptosis, steroid biosynthesis, glycosaminoglycan degradation, glycolysis/gluconeogenesis, and the lysosome. (Figure 4A and Table S4). For the CKb vs. Tb comparison, 24 DEGs were enriched in the 32 KEGG pathways, among which 13 pathways (40.6%) belonged to metabolism pathways, and the main enrichment pathways contained herpes simplex infection, ascorbate and aldarate metabolism, fatty acid biosynthesis, drug metabolism—other enzymes, and ABC transporters (Figure 4B and Table S4). These results indicated that the JHA treatment affected mainly the metabolism pathways in the *G. daurica* adults.

The GO enrichment analysis indicated that for the CKa vs. Ta comparison, 20, nine, and three terms were significantly enriched in the biological process, cellular component, and molecular function, respectively. The top three most significantly terms were the oxidation–reduction process, nucleic acid metabolic process, and developmental process for the biological process; ribonucleoprotein complex, cytoplasm, and cell periphery for the cellular component; and nucleic acid binding, oxidoreductase activity, and catalytic activity for the molecular function. For the CKb vs. Tb comparison, 32, 12, and six terms were significantly enriched in the biological process, cellular component, and molecular function, respectively. The top three most significantly enriched terms were translation, ribosome assembly, and the oxidation–reduction process for the biological process; ribosome, intracellular organelle lumen, and ribosomal subunit for the cellular component; and nucleic acid binding, oxidoreductase activity, and structural constituent of cuticle for the molecular function (Table S5).

### 3.4. Effects of JHA Application on JH Signaling Pathway-Related Gene Expression

**Figure 5:**
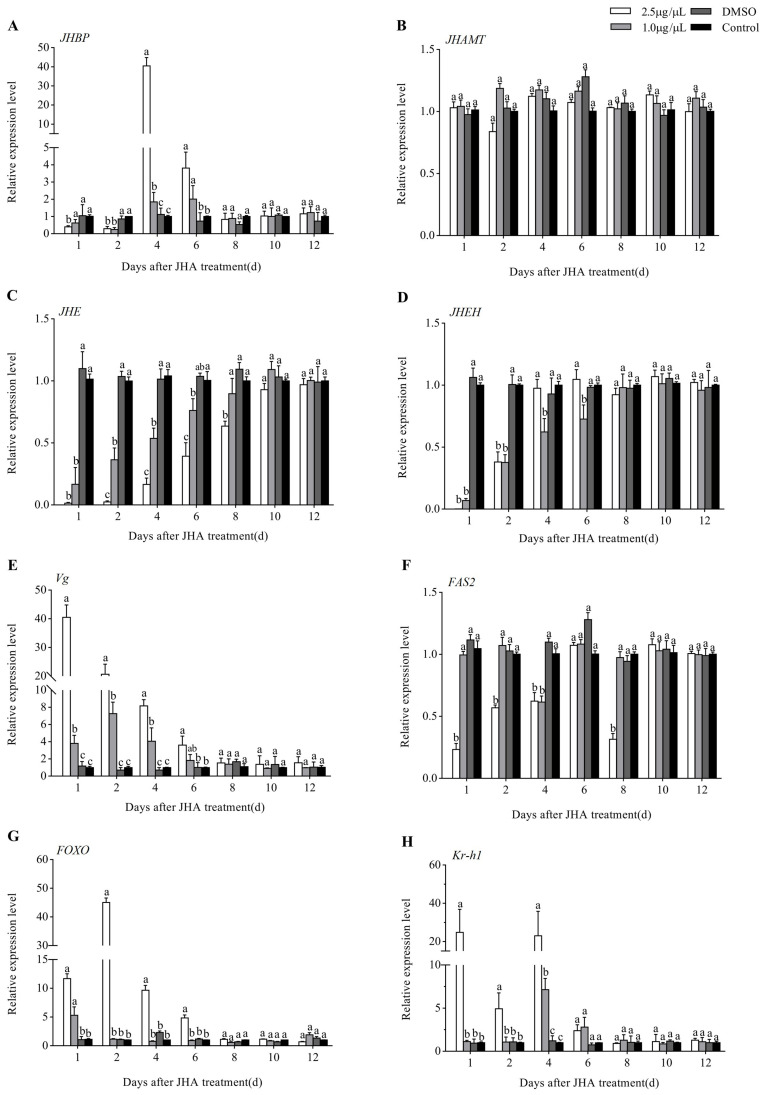
Expression profiles of 8 JH signaling-related genes after JHA application at 3 days post-eclosion. Different letters above the columns indicate significant difference among different treatments at the same time at *p* < 0.05 (Tukey’s HSD test). (**A**): *JHBP*; (**B**): *JHAMP*; (**C**): *JHE*; (**D**): *JHEH*; (**E**): *Vg*; (**F**): *FAS2*; (**G**): *FOXO*; (**H**): *Kr-h1*.

To reveal the molecular mechanism of JHA inhibiting reproductive diapause, the relative expression profiles of the eight JH signaling-related DEGs, including *JHAMT*, *JHBP*, *JHE*, *JHEH*, *Kr-h1*, *FOXO*, *Vg*, and *FAS2*, were further analyzed by qRT-PCR at more time points after JHA treatment. We first examined the effects of JHA application at 3 days post adult eclosion. qRT-PCR analysis showed that both two doses (2.5 and 1.0 μg/μL methoprene) of JHA application influenced significantly the expression of all the other seven genes except for *JHAMT* (*p* < 0.05) (Figure 5). Compared with the DMSO-treated or untreated controls, *JHBP* was down-regulated during the first 2 days after treatment but up-regulated at 4 and 6 days after treatment (Figure 5A). JHA treatments decreased the *JHE* expression from 1 to 6 days at a low dose (1.0 μg/μL) or 8 days at a high dose (2.5 μg/μL) after treatment (Figure 5C). The *Vg* expression was up-regulated from 1 to 4 days (1.0 μg/μL) or 6 days (2.5 μg/μL) after treatment, and the stimulating effect at a dose of 2.5 μg/μL was much higher than at a dose of 1.0 μg/μL (Figure 5E). JHA application down-regulated significantly the expression of *FAS2* only at a high dose of 2.5 μg/μL. The high-dose treatment induced the expression of *FOXO* from 1 to 6 days after treatment whereas the low dose did only at the first day (Figure 5G). *Kr-h1* showed a similar response to methoprene treatment to *FOXO* (Figure 5H). However, the *JHEH* expression was reduced during the first 6 days after treatment with a low dose of 1.0 μg/μL whereas this inhibiting effect lasted for 2 days after treatment with a high dose of 2.5 μg/μL (Figure 5D).

**Figure 6:**
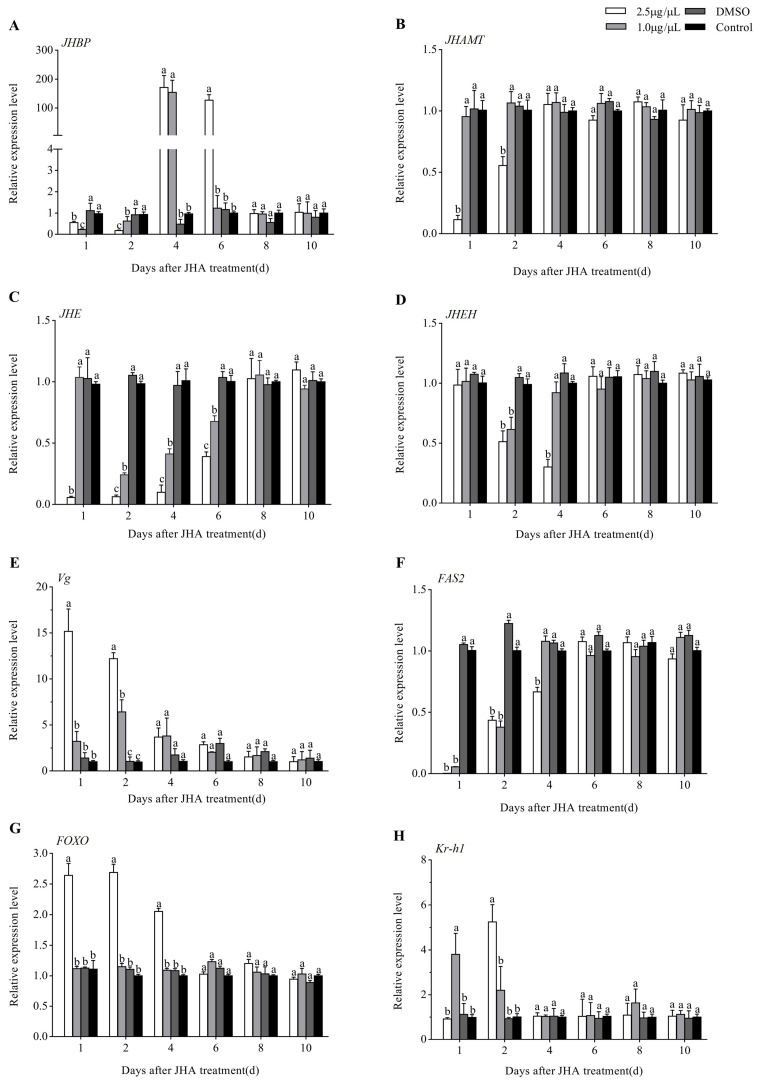
Expression profiles of 8 JH signaling-related genes after JHA application at 5 days post-eclosion. Different letters above the columns indicate significant difference among different treatments at the same time at *p* < 0.05 (Tukey’s HSD test). (**A**): *JHBP*; (**B**): *JHAMP*; (**C**): *JHE*; (**D**): *JHEH*; (**E**): *Vg*; (**F**): *FAS2*; (**G**): *FOXO*; (**H**): *Kr-h1*.

Figure 6 showed that the effects of JHA application at 5 days post adult eclosion were similar to those at 3 days post adult eclosion except for *JHAMT*, which was down-regulated during 2 days after treatment at a high dose of 2.5 μg/μL. In conclusion, JHA methoprene could promote the expression of *Vg*, *FOXO* and *Kr-h1* while suppressing the expression of *JHBP*, *JHAMT*, *JHE*, *JHEH* and *FAS2*, and the action strength increased with the JHA dose increasing except for *JHEH*. However, JHA application during diapause (at 15 days post adult eclosion) did not significantly affect the expression of the above eight genes, in keeping with no influence on the diapause rates of the *G. daurica* adults (Figure S1).

## 4. Discussion

A great deal of literature shows that the lack of JH can lead to reproductive diapause in insects [4]. Schooneveld et al. [13] first reported that the topical application of JH only postponed diapause during pre-diapause of *L. decemlineata*, but adults during diapause were activated rather easily. JHA treatment (methoprene) could terminate reproductive diapause of *M. pusilla* [15]. JH treatment restrained diapause in *Culex tritaeniorhynchus* whereas it promoted the diapausing females to terminate diapause [32]. Hiroyoshi et al. [16] also found that JHA, methoprene, promoted the development of ovaries and of the male accessory glands and simplex, indicating that methoprene could induce the termination of reproductive diapause. However, in this study, JHA treatment in the pre-diapause stage could delay temporarily the *G. daurica* adults entering diapause but could not break diapause when JHA was applied during diapause. A possible reason is that the other aforementioned insect species are species with facultative diapause whereas diapause in *G. daurica* belongs to obligatory diapause, which can be usually terminated only after a longer diapause maintenance period.

In the present study, the regulation network of JH signaling-associated genes in *G. daurica* was investigated through the transcriptome sequencing after JHA treatment. The results showed that a great number of genes changed at the transcriptional expression level, and these DEGs were enriched in various metabolism pathways, suggesting that JH might function in the regulation of diapause by regulating metabolic processes in *G. daurica* [33]. Zhu et al. [34] also found that mRNA levels of 16 and 72 genes were enhanced at 3 and 12 h after JH treatment using microarray hybridization meanwhile mRNA levels of 33 and 76 genes were significantly suppressed at 3 and 12 h, respectively, in newly emerged adults of *Aedes aegypti*, and the observed changes could be also induced by the JHAs, methoprene and pyriproxyfen, but not by farnesol.

In this study, the mRNA levels of several key genes involved in JH biosynthesis and degradation processes were found to change after JHA application, such as *JHAMT*, *JHE*, *JHEH*, and *JHBP*. JH titer in insects is regulated by JH biosynthesis and degradation. It has been reported that JHE and *JHEH* in hemolymph are involved in the JH degradation [34]. We found that topical treatment with JHA methoprene could suppress the expression of *JHE* and *JHEH* in *G. daurica*. Similarly, the JH mimic pyriproxyfen significantly repressed the *JHE* transcription during the diapause induction phase of *L. decemlineata*, confirming the important role of JHE in diapause induction of *L. decemlineata* [35]. In contrast, exogenous JHA treatment promoted the expression of *JHE* in *A. aegypti* [34], *Heliothis virescens* [36], *Plutella xylostella* [37,38], and *Colaphellus bowringi* [39]). In *Neocaridina davidi*, *JHEH* was up-regulated after JHA treatment and this result could be caused by the hormonal feedback system [40]. *JHAMT* and *JHBP* are the most important regulators in the process of JH biosynthesis and transportation [41,42]. Our qRT-PCR results showed that *JHBP* was down-regulated in the first two days after JHA treatment, but up-regulated at 4 and 6 days after JHA treatment in *G. daurica*. However, *JHBP* was up-regulated following JHA treatment (methoprene) in other insects, such as *Omphisa fuscidentalis* [43] and *Helicoverpa armigera* [44]. Additionally, JHA application inhibited the expression of *JHAMT* in *G. daurica*, in agreement with the observation in *C. bowringi* [45]. The above results show that the genes involved in the regulation of JH exhibit obvious differences between various insect species, which indicates that the mechanism of hormone activity in insects is very complicated and the regulation of JH biosynthesis and degradation is variable. Our work further verifies that the application of exogenous JHA can directly regulate the expression of the JH-signaling pathway genes, and it has great potential in the control of diapause and reproduction of *G. daurica*.

Kr-h1, a zinc finger transcription factor, plays a key downstream role in the JH pathway as a transductor of JH activity [46]. Kr-h1 has been found to be involved in the regulation of metamorphosis, vitellogenesis and oocyte maturation [47,48,49,50,51,52,53]. Stagnation in reproduction is a key feature of adult (reproductive) diapause, which implies an arrest in oocyte development for females [4]. Therefore, Kr-h1 may function in regulating adult diapause. In this study, JHA treatment was found to induce the expression of *Kr-h1* and *Vg*. Yue et al. [49] also found that JHA treatment (methoprene) significantly increased the expression of *Kr-h1* and *Vg*, and accelerated ovary development in *Bactrocera dorsalis*. Topical application of JH III stimulated the expression of *Kr-h1* in *Sitodiplosis mosellana* [54]. The expression of *Vg* induced through treatment with JH or JHA was also found in other insects, such as *Blattella germanica* [55], *Tribolium castaneum* [56], *Pyrrhocoris apterus* [57], and *C. bowringi* [45].

The insulin signaling pathway and its downstream target, FOXO, play an important role in regulating diapause in some insects, such as *Drosophila melanogaster* [58], *Culex pipiens* [59] and *Laodelphax striatellus* [60]. In the present study, JHA treatment enhanced the expression of *FOXO* and *Vg*, and postponed the initiation of adult diapause. It was also reported that knockdown of *FOXO* by RNAi inhibited the *Vg* expression in *A. aegypti* [61], and led to a significantly extended duration of summer diapause pupae in *Delia antiqua* [62]. Nevertheless, topical application of JH III to diapause-destined females of *C. pipiens* suppressed the FOXO protein expression [63]. This difference might be due to different diapause forms—obligatory summer diapause in *G. daurica* whereas facultative winter in *C. pipiens*. We speculate that two transcription factors, Kr-h1 and FOXO, might also play important roles in regulating summer diapause of *G. daurica* adults.

After JHA treatment, many DEGs were enriched in various metabolism pathways which took up 50% and 40.6% of all enriched pathways for the DEGs at 1 and 2 days post-JHA treatment, respectively (Table S4). Trehalose transporters (TRETs) participate in transferring trehalose from the fat body into the hemolymph, and they have been also found to play an important role in insect growth and energy metabolism [64]. Although trehalose has been shown to contribute to diapause and reproduction, whether TRETs play roles in these processes remains unclear [65]. In this study, two *TRET1* genes and one *TRET1* gene were differentially down-regulated 1 and 2 days, respectively, after JHA application. Li et al. [65] also recently reported that JHA induced *TRET1b* but repressed *TRET1a* at transcriptional levels in *C. bowringi*. Fatty acid biosynthesis and metabolism were common enriched pathways (Figure 4). JHA treatment also inhibited the *FAS2* expression, in keeping with the decreasing of the lipid content in *G. daurica*. Tan et al., got the same results in *C. bowringi* [19]. These results show that JH signaling plays a regulatory role in the fatty acid biosynthesis and metabolism. However, a transcript (c82613.graph_c0) coding fatty acid synthase-like was up-regulated after JHA application (Table 1). We speculate that JH may have different effects on different FASs, which needs further investigation.

## 5. Conclusions

JHA application postponed diapause initiation and inhibited the lipid accumulation when the treatment was performed at the pre-diapause stage, whereas it did not affect diapause when the treatment was conducted during diapause in *G. daurica*. JHA application affected mainly the metabolism pathways. JHA could promote the expression of *Vg*, *FOXO* and *Kr-h1* while it suppresses the expression of *JHBP*, *JHAMT*, *JHE*, *JHEH* and *FAS2*, and the action strength increased with the JHA dose increasing. These results show that JH signaling plays an important role in the regulation of reproductive summer diapause in *G. daurica*.

## Figures and Tables

**Table 1:** Common 11 differentially expressed genes in CKa vs. Ta and CKb vs. Tb comparisons.

DEG_ID	Cka vs. Ta			CKb vs. Tb			Nr_Annotation
	FDR	Log2FC	Regulated	FDR	Log2FC	Regulated	
c85279.graph_c0	2.37 × 10^−13^	2.575	up	2.92 × 10^−2^	1.303	up	vitellogenin-like
c82613.graph_c0	6.25 × 10^−6^	1.369	up	1.19 × 10^−3^	1.380	up	fatty acid synthase-like
c74827.graph_c0	3.39 × 10^−3^	1.336	up	5.83 × 10^−3^	1.558	up	uncharacterized protein LOC111513065
c71191.graph_c0	1.61 × 10^−5^	1.272	up	4.84 × 10^−3^	1.228	up	probable pseudouridine-5&apos;-phosphatase
c69643.graph_c0	8.60 × 10^−3^	−1.007	down	7.39 × 10^−4^	−1.295	down	retinoid-inducible serine carboxypeptidase-like
c37561.graph_c0	2.11 × 10^−6^	−1.034	down	1.46 × 10^−9^	−1.617	down	PREDICTED: crustapain-like
c64926.graph_c0	5.25 × 10^−3^	−1.065	down	7.01 × 10^−11^	−1.777	down	--
c84589.graph_c0	9.74 × 10^−5^	−1.160	down	1.64 × 10^−5^	−1.526	down	protein msta-like
c72112.graph_c0	1.80 × 10^−5^	−1.360	down	6.99 × 10^−30^	−2.327	down	facilitated trehalose transporter Tret1-like
c73343.graph_c0	2.44 × 10^−3^	−1.365	down	4.20 × 10^−2^	−1.330	down	--
c82886.graph_c0	3.61 × 10^−6^	−1.401	down	4.31 × 10^−2^	−1.235	down	nose resistant to fluoxetine protein 6-like isoform X1

## Data Availability

Data is contained in the article and Supplementary Materials.

## Supplementary Materials

The following are available online at https://www.mdpi.com/2075-4450/12/3/237/s1, Figure S1: Expression profiles of 8 JH signaling-related genes after JHA application at 15 days post eclosion, Table S1: Primers for qPCR used in this study, Table S2: Assembly result statistics table, Table S3: The annotation and *p*-value of DEGs in the transcriptomes, Table S4: Enriched KEGG pathways of DEGs, Table S5: Significantly enriched GO terms for the DEGs.
