# Supplementary material for: Regulation of Juvenile Hormone on Summer Diapause of Geleruca daurica and Its Pathway Analysis
Source: Insects. 2021 Mar 11;12(3):237. doi: 10.3390/insects12030237 (PMC8000908; doi:10.3390/insects12030237)
Supplement: Supplementary file 1 [file insects-12-00237-s001.zip › insects-1110083-suppl-update/Table S1.docx]

| **Table S1. Primers for qPCR used in this study** | |
| --- | --- |
| Primers | Sequence for qPCR (5-3) |
| JHE-F | ACCGTTTTTGGACAAAGTGC |
| JHE-R | AATGCTTCACGTTCCGTCTT |
| JHBP-F | GCCACTTTCAACGAGCTAGG |
| JHBP-R | TGAATTCCACCTCCGAAAAG |
| JHEH-F | AACCGCGCCAATACAGAAAA |
| JHEH-R | TGGAATGCAGCGAAATGTCC |
| JHAMT-F | AGTGCTGTGAATCCCATTGT |
| JHAMT-R | GGTTTCGAAGCAACAACGAT |
| Vg-F | CCCAAGCTGCATACTTGTTG |
| Vg-R | TCGATTTGGTCTTCGGATTC |
| Met-F | CGGATGCGATCAAAGAATTT |
| Met-R | CCCTGGTCGTCTATTTCCAA |
| FOXO-F | ACAACCGACACAACAGACCA |
| FOXO-R | TCCTGTATGAGGCTCGCTTT |
| Kr-h1-F | TGGATACAACCACGTCCTCA |
| Kr-h1-R | TCTTGAACGAGGGGTAATGG |
| FAS-F | TCATCTTCCCGCTCCAGTTT |
| FAS-R | GCGACATCATGGGCATTGAT |
| SDHA-F | GGGAGACCACCATCTCCTCA |
| SDHA-R | AGCTGGTGCTCCTAAGTCCA |
