# Supplementary material for: Regulation of Juvenile Hormone on Summer Diapause of Geleruca daurica and Its Pathway Analysis
Source: Insects. 2021 Mar 11;12(3):237. doi: 10.3390/insects12030237 (PMC8000908; doi:10.3390/insects12030237)
Supplement: Supplementary file 1 [file insects-12-00237-s001.zip › insects-1110083-suppl-update/Table S2.docx]

| **Table S2.** Assembly result statistics table | | |
| --- | --- | --- |
| Length Range | Transcript | Unigene |
| 200-300 | 47,415(23.36%) | 33,372(38.25%) |
| 300-500 | 36,330(17.90%) | 19,686(22.57%) |
| 500-1000 | 38,955(19.19%) | 14,473(16.59%) |
| 1000-2000 | 40,004(19.71%) | 10,851(12.44%) |
| 2000+ | 40,270(19.84%) | 8,854(10.15%) |
| Total Number | 202,974 | 87,236 |
| Total Length | 260,183,935 | 73,114,549 |
| N50 Length | 2,378 | 1,677 |
| Mean Length | 1281.86 | 838.12 |
