# Supplementary material for: Regulation of Juvenile Hormone on Summer Diapause of Geleruca daurica and Its Pathway Analysis
Source: Insects. 2021 Mar 11;12(3):237. doi: 10.3390/insects12030237 (PMC8000908; doi:10.3390/insects12030237)
Supplement: Supplementary file 1 [file insects-12-00237-s001.zip › insects-1110083-suppl-update/Table S3.docx]

| **Table S3. The annotation and P-alue of DEGs in the transcriptome** | | | | |
| --- | --- | --- | --- | --- |
| DEGs_id | P-value | log2FC | regulated | nr_annotation |
| Cka vs Ta |  |  |  |  |
| c85279.graph_c0 | 4.84E-17 | 2.5745 | up | vitellogenin-like [Leptinotarsa decemlineata] |
| c66901.graph_c0 | 1.36E-35 | 1.9922 | up | chemosensory protein [Galeruca daurica] |
| c83339.graph_c0 | 2.93E-10 | 1.9577 | up | vitellogenin-like [Leptinotarsa decemlineata] |
| c80223.graph_c0 | 1.33E-10 | 1.5519 | up | Krueppel homolog 1-like isoform X1 [Anoplophora glabripennis] |
| c66641.graph_c0 | 1.12E-06 | 1.4963 | up | PREDICTED: uncharacterized protein LOC106118770, partial [Papilio xuthus] |
| c73192.graph_c0 | 2.22E-12 | 1.4567 | up | protein yellow [Leptinotarsa decemlineata] |
| c88308.graph_c0 | 2.67E-07 | 1.3775 | up | uncharacterized protein LOC105841937 [Bombyx mori] |
| c82613.graph_c0 | 1.05E-08 | 1.3694 | up | fatty acid synthase-like [Leptinotarsa decemlineata] |
| c74827.graph_c0 | 1.76E-05 | 1.3364 | up | uncharacterized protein LOC111513065 [Leptinotarsa decemlineata] |
| c82978.graph_c0 | 4.28E-07 | 1.2938 | up | None |
| c79893.graph_c0 | 4.29E-10 | 1.2844 | up | sterol O-acyltransferase 1-like [Anoplophora glabripennis] |
| c71191.graph_c0 | 3.36E-08 | 1.2716 | up | probable pseudouridine-5&apos;-phosphatase [Leptinotarsa decemlineata] |
| c85424.graph_c0 | 1.84E-05 | 1.2482 | up | thaumatin-like protein 1 [Leptinotarsa decemlineata] |
| c74611.graph_c0 | 6.73E-05 | 1.2292 | up | None |
| c76727.graph_c0 | 1.75E-14 | 1.1990 | up | phosphoenolpyruvate carboxykinase [GTP] isoform X1 [Anoplophora glabripennis] |
| c61803.graph_c0 | 2.55E-11 | 1.1946 | up | PREDICTED: endocuticle structural glycoprotein SgAbd-8-like [Nicrophorus vespilloides] |
| c82134.graph_c0 | 5.72E-06 | 1.1696 | up | uncharacterized protein LOC108906439 [Anoplophora glabripennis] |
| c57572.graph_c0 | 1.62E-04 | 1.1679 | up | RecName: Full=Coleoptericin |
| c72461.graph_c1 | 1.19E-04 | 1.1533 | up | None |
| c84509.graph_c1 | 1.53E-07 | 1.1488 | up | PREDICTED: aldose reductase [Tribolium castaneum] |
| c80710.graph_c0 | 1.13E-09 | 1.1448 | up | beta-glucuronidase-like protein [Leptinotarsa decemlineata] |
| c76685.graph_c0 | 8.01E-11 | 1.1441 | up | esterase [Leptinotarsa decemlineata] |
| c65621.graph_c0 | 1.63E-04 | 1.1199 | up | None |
| c68944.graph_c0 | 2.35E-05 | 1.1198 | up | cuticle protein 38-like [Leptinotarsa decemlineata] |
| c39745.graph_c0 | 4.99E-04 | 1.0833 | up | None |
| c87745.graph_c0 | 2.62E-04 | 1.0811 | up | lachesin-like, partial [Leptinotarsa decemlineata] |
| c38485.graph_c0 | 1.35E-20 | 1.0669 | up | retinol-binding protein pinta [Anoplophora glabripennis] |
| c64382.graph_c0 | 3.60E-04 | 1.0631 | up | PREDICTED: sporulation-specific protein 15-like, partial [Hyalella azteca] |
| c89602.graph_c0 | 1.70E-06 | 1.0520 | up | male-specific doublesex isoform m isoform 2 [Tribolium castaneum] |
| c80049.graph_c0 | 6.67E-05 | 1.0487 | up | putative fatty acyl-CoA reductase CG5065 [Leptinotarsa decemlineata] |
| c80249.graph_c0 | 3.47E-06 | 1.0471 | up | None |
| c80392.graph_c1 | 8.77E-05 | 1.0458 | up | Uncharacterized protein OBRU01_09660 [Operophtera brumata] |
| c48341.graph_c0 | 4.51E-04 | 1.0424 | up | None |
| c62194.graph_c0 | 1.41E-08 | 1.0415 | up | None |
| c75690.graph_c1 | 2.47E-15 | 1.0308 | up | actin-binding Rho-activating protein-like isoform X2 [Leptinotarsa decemlineata] |
| c75172.graph_c0 | 9.52E-04 | 1.0252 | up | hypothetical protein ALC60_00999, partial [Trachymyrmex zeteki] |
| c84682.graph_c0 | 1.50E-05 | 1.0120 | up | exocyst complex component 4 [Anoplophora glabripennis] |
| c88407.graph_c0 | 1.03E-03 | 0.9948 | up | RNA-directed DNA polymerase from mobile element jockey-like isoform X1 [Papilio machaon] |
| c86752.graph_c0 | 7.73E-04 | 0.9719 | up | integrase core domain protein [Lasius niger] |
| c86564.graph_c0 | 6.91E-17 | 0.9681 | up | None |
| c77337.graph_c0 | 1.11E-03 | 0.9580 | up | predicted protein [Nematostella vectensis] |
| c78019.graph_c0 | 8.11E-06 | 0.9566 | up | cytochrome c [Zootermopsis nevadensis] |
| c84618.graph_c0 | 4.68E-04 | 0.9549 | up | None |
| c83803.graph_c0 | 1.25E-03 | 0.9454 | up | None |
| c50095.graph_c0 | 1.19E-03 | 0.9388 | up | None |
| c71732.graph_c0 | 2.52E-03 | 0.9348 | up | alpha-tocopherol transfer protein-like [Anoplophora glabripennis] |
| c67906.graph_c0 | 2.01E-03 | 0.9327 | up | None |
| c80891.graph_c0 | 1.81E-06 | 0.9323 | up | aminopeptidase N-like [Leptinotarsa decemlineata] |
| c83852.graph_c0 | 7.50E-07 | 0.9267 | up | toll-like receptor Tollo isoform X2 [Leptinotarsa decemlineata] |
| c70800.graph_c0 | 1.18E-03 | 0.9255 | up | PREDICTED: origin recognition complex subunit 1-like, partial [Papilio xuthus] |
| c80814.graph_c0 | 7.07E-05 | 0.9249 | up | None |
| c52530.graph_c0 | 6.56E-04 | 0.9239 | up | hypothetical protein DDB_G0288781 [Dictyostelium discoideum AX4] |
| c73442.graph_c0 | 6.89E-09 | 0.9226 | up | protein hairy [Anoplophora glabripennis] |
| c88261.graph_c0 | 2.05E-05 | 0.9154 | up | facilitated trehalose transporter Tret1-like isoform X1 [Leptinotarsa decemlineata] |
| c77387.graph_c0 | 2.52E-03 | 0.9154 | up | xenotropic and polytropic retrovirus receptor 1 [Aedes aegypti] |
| c73402.graph_c0 | 1.44E-06 | 0.9152 | up | None |
| c18657.graph_c0 | 1.68E-03 | 0.9135 | up | None |
| c50681.graph_c0 | 5.57E-05 | 0.9005 | up | carboxypeptidase N subunit 2-like [Centruroides sculpturatus] |
| c79476.graph_c0 | 2.06E-03 | 0.8977 | up | -- |
| c78368.graph_c0 | 9.19E-05 | 0.8905 | up | serine protease 7-like [Anoplophora glabripennis] |
| c80171.graph_c0 | 1.51E-04 | 0.8897 | up | prostaglandin E2 receptor EP2 subtype isoform X2 [Leptinotarsa decemlineata] |
| c82544.graph_c0 | 1.65E-03 | 0.8881 | up | None |
| c71239.graph_c0 | 2.00E-03 | 0.8832 | up | -- |
| c74285.graph_c0 | 2.38E-03 | 0.8820 | up | None |
| c86495.graph_c0 | 9.98E-04 | 0.8804 | up | uncharacterized protein LOC105841287 isoform X1 [Bombyx mori] |
| c73524.graph_c0 | 1.50E-03 | 0.8801 | up | PREDICTED: forkhead box protein A2-A-like [Acropora digitifera] |
| c56742.graph_c0 | 2.47E-03 | 0.8774 | up | None |
| c79002.graph_c0 | 4.41E-03 | 0.8694 | up | facilitated trehalose transporter Tret1-like [Leptinotarsa decemlineata] |
| c63388.graph_c0 | 2.64E-04 | 0.8663 | up | alpha-N-acetylgalactosaminidase [Anoplophora glabripennis] |
| c86778.graph_c0 | 1.74E-08 | 0.8642 | up | uncharacterized protein LOC111511541 [Leptinotarsa decemlineata] |
| c48915.graph_c0 | 2.45E-03 | 0.8634 | up | PREDICTED: uncharacterized protein LOC106115104 [Papilio xuthus] |
| c88560.graph_c0 | 5.94E-07 | 0.8627 | up | ankyrin-3-like isoform X1 [Leptinotarsa decemlineata] |
| c83287.graph_c1 | 6.58E-06 | 0.8596 | up | multidrug resistance-associated protein 4 [Anoplophora glabripennis] |
| c79582.graph_c0 | 2.26E-05 | 0.8594 | up | UDP-glucuronosyltransferase 2C1-like [Leptinotarsa decemlineata] |
| c74881.graph_c0 | 3.07E-03 | 0.8564 | up | Kinesin-like protein KIF18B [Trichinella murrelli] |
| c83757.graph_c0 | 3.28E-03 | 0.8552 | up | PREDICTED: DNA-directed RNA polymerase I subunit RPA1 isoform X2 [Diuraphis noxia] |
| c81997.graph_c0 | 1.74E-03 | 0.8509 | up | pyroglutamylated RFamide peptide receptor-like [Anoplophora glabripennis] |
| c88219.graph_c0 | 9.72E-05 | 0.8492 | up | uncharacterized protein LOC110996927 isoform X1 [Pieris rapae] |
| c82816.graph_c0 | 3.55E-10 | 0.8491 | up | endonuclease-reverse transcriptase [Bombyx mori] |
| c67706.graph_c0 | 3.84E-03 | 0.8476 | up | None |
| c75733.graph_c0 | 2.05E-05 | 0.8446 | up | farnesol dehydrogenase-like [Leptinotarsa decemlineata] |
| c85832.graph_c0 | 8.80E-04 | 0.8392 | up | copia protein, partial [Lasius niger] |
| c86287.graph_c0 | 6.58E-03 | 0.8377 | up | uncharacterized protein LOC111513283 [Leptinotarsa decemlineata] |
| c68574.graph_c0 | 3.48E-03 | 0.8332 | up | -- |
| c59106.graph_c0 | 5.23E-06 | 0.8329 | up | PREDICTED: caltractin-like isoform X1 [Agrilus planipennis] |
| c69566.graph_c0 | 1.06E-03 | 0.8321 | up | uncharacterized protein LOC108905900 [Anoplophora glabripennis] |
| c63581.graph_c0 | 3.80E-03 | 0.8306 | up | structural maintenance of chromosomes protein 4 [Ceratitis capitata] |
| c85471.graph_c0 | 1.25E-03 | 0.8304 | up | tumor necrosis factor receptor superfamily member wengen [Leptinotarsa decemlineata] |
| c72058.graph_c0 | 1.77E-04 | 0.8275 | up | PREDICTED: uncharacterized protein LOC108776222 [Cyphomyrmex costatus] |
| c62023.graph_c0 | 3.71E-03 | 0.8252 | up | la protein homolog [Limulus polyphemus] |
| c57035.graph_c0 | 3.88E-03 | 0.8195 | up | None |
| c84855.graph_c1 | 7.04E-03 | 0.8177 | up | None |
| c80218.graph_c0 | 5.52E-03 | 0.8163 | up | lysosomal acid phosphatase [Anoplophora glabripennis] |
| c88133.graph_c0 | 2.31E-03 | 0.8131 | up | None |
| c75199.graph_c0 | 1.05E-04 | 0.8094 | up | catalase-like [Anoplophora glabripennis] |
| c80762.graph_c0 | 5.28E-07 | 0.8083 | up | None |
| c69503.graph_c0 | 4.40E-03 | 0.8074 | up | histone deacetylase 3-like [Stylophora pistillata] |
| c90161.graph_c0 | 9.96E-03 | 0.8013 | up | None |
| c71873.graph_c0 | 5.06E-03 | 0.7986 | up | None |
| c80413.graph_c0 | 4.68E-03 | 0.7980 | up | None |
| c80907.graph_c0 | 9.87E-03 | 0.7972 | up | PREDICTED: uncharacterized protein LOC101238582 [Hydra vulgaris] |
| c86124.graph_c0 | 4.12E-06 | 0.7969 | up | phospholipase D3-like isoform X2 [Leptinotarsa decemlineata] |
| c83075.graph_c0 | 5.07E-03 | 0.7962 | up | facilitated trehalose transporter Tret1-like [Leptinotarsa decemlineata] |
| c77042.graph_c0 | 7.92E-03 | 0.7960 | up | zinc finger protein 585A [Anoplophora glabripennis] |
| c82716.graph_c0 | 1.04E-05 | 0.7959 | up | None |
| c83089.graph_c0 | 6.25E-03 | 0.7954 | up | uncharacterized protein LOC108906385 [Anoplophora glabripennis] |
| c81343.graph_c0 | 5.01E-03 | 0.7944 | up | PREDICTED: structural maintenance of chromosomes protein 6-like [Papilio xuthus] |
| c77483.graph_c0 | 3.22E-05 | 0.7942 | up | lipoate protein ligase [Phyllotreta striolata] |
| c73653.graph_c0 | 5.52E-03 | 0.7880 | up | PREDICTED: 25S rRNA (cytosine-C(5))-methyltransferase nop2-like [Papilio xuthus] |
| c47646.graph_c1 | 6.13E-03 | 0.7872 | up | -- |
| c88304.graph_c0 | 1.04E-05 | 0.7851 | up | PREDICTED: uncharacterized protein LOC105556050 [Vollenhovia emeryi] |
| c84341.graph_c0 | 5.45E-03 | 0.7837 | up | None |
| c54113.graph_c0 | 2.50E-03 | 0.7813 | up | odorant-binding protein [Galeruca daurica] |
| c78725.graph_c0 | 6.91E-03 | 0.7747 | up | None |
| c72327.graph_c0 | 6.34E-03 | 0.7731 | up | None |
| c65940.graph_c0 | 6.57E-03 | 0.7731 | up | None |
| c79474.graph_c0 | 6.80E-03 | 0.7727 | up | hypothetical protein SteCoe_19010 [Stentor coeruleus] |
| c80095.graph_c0 | 3.57E-05 | 0.7717 | up | maltase 2-like isoform X2 [Leptinotarsa decemlineata] |
| c62216.graph_c0 | 6.84E-03 | 0.7695 | up | None |
| c66757.graph_c0 | 7.88E-03 | 0.7674 | up | PREDICTED: glycylpeptide N-tetradecanoyltransferase 2-like [Papilio xuthus] |
| c79621.graph_c0 | 2.47E-04 | 0.7660 | up | uncharacterized protein LOC111514166 isoform X2 [Leptinotarsa decemlineata] |
| c54408.graph_c0 | 6.72E-03 | 0.7657 | up | PREDICTED: mRNA cap guanine-N7 methyltransferase-like [Papilio xuthus] |
| c88869.graph_c0 | 5.00E-03 | 0.7640 | up | Retrovirus-related Pol polyprotein from transposon 17.6, partial [Stegodyphus mimosarum] |
| c48807.graph_c0 | 7.44E-03 | 0.7632 | up | PREDICTED: uncharacterized protein LOC106115193 [Papilio xuthus] |
| c81874.graph_c0 | 2.89E-03 | 0.7623 | up | None |
| c39350.graph_c0 | 9.25E-03 | 0.7622 | up | PREDICTED: 60S ribosomal protein L12-like [Papilio xuthus] |
| c65120.graph_c0 | 1.11E-03 | 0.7618 | up | hypothetical protein YQE_10283, partial [Dendroctonus ponderosae] |
| c65594.graph_c0 | 4.22E-04 | 0.7600 | up | elongation of very long chain fatty acids protein AAEL008004 isoform X4 [Ceratitis capitata] |
| c62209.graph_c0 | 7.63E-03 | 0.7582 | up | None |
| c70266.graph_c0 | 6.79E-03 | 0.7565 | up | PREDICTED: bromodomain testis-specific protein-like [Papilio xuthus] |
| c54976.graph_c0 | 7.11E-03 | 0.7556 | up | -- |
| c83399.graph_c0 | 7.55E-03 | 0.7537 | up | sodium-coupled neutral amino acid transporter 9 homolog [Anoplophora glabripennis] |
| c82443.graph_c0 | 9.00E-06 | 0.7528 | up | glutamic acid-rich protein-like isoform X1 [Leptinotarsa decemlineata] |
| c66046.graph_c0 | 5.70E-03 | 0.7518 | up | -- |
| c89816.graph_c0 | 2.34E-03 | 0.7487 | up | PREDICTED: uncharacterized protein LOC105556767, partial [Vollenhovia emeryi] |
| c72010.graph_c0 | 8.34E-03 | 0.7465 | up | PREDICTED: aminopeptidase N-like isoform X3 [Halyomorpha halys] |
| c77070.graph_c0 | 5.87E-04 | 0.7393 | up | protein peste-like [Anoplophora glabripennis] |
| c81788.graph_c0 | 2.22E-03 | 0.7384 | up | None |
| c87855.graph_c0 | 5.35E-03 | 0.7384 | up | RNA-directed DNA polymerase from mobile element jockey, partial [Stegodyphus mimosarum] |
| c62432.graph_c0 | 8.40E-03 | 0.7383 | up | PREDICTED: 26S proteasome non-ATPase regulatory subunit 12-like [Papilio xuthus] |
| c25973.graph_c0 | 5.56E-03 | 0.7359 | up | -- |
| c85710.graph_c0 | 2.80E-07 | 0.7344 | up | 6-phosphofructo-2-kinase/fructose-2,6-bisphosphatase-like [Leptinotarsa decemlineata] |
| c81402.graph_c0 | 2.19E-03 | 0.7317 | up | antichymotrypsin-2-like [Leptinotarsa decemlineata] |
| c84413.graph_c0 | 5.29E-03 | 0.7301 | up | hypothetical protein BSL78_04670 [Apostichopus japonicus] |
| c67335.graph_c0 | 1.07E-05 | 0.7299 | up | uncharacterized protein LOC108908510 [Anoplophora glabripennis] |
| c79213.graph_c1 | 1.31E-07 | 0.7287 | up | sestrin homolog [Anoplophora glabripennis] |
| c79216.graph_c0 | 5.65E-05 | 0.7282 | up | ABC transporter G family member 20 isoform X4 [Anoplophora glabripennis] |
| c77595.graph_c0 | 1.00E-08 | 0.7267 | up | protein patched homolog 1 isoform X1 [Anoplophora glabripennis] |
| c78420.graph_c0 | 1.98E-05 | 0.7265 | up | uncharacterized protein LOC108910200 [Anoplophora glabripennis] |
| c58139.graph_c0 | 7.95E-04 | 0.7239 | up | pseudouridine-metabolizing bifunctional protein C1861.05 [Anoplophora glabripennis] |
| c71380.graph_c0 | 9.27E-03 | 0.7201 | up | hypothetical protein AMSG_01929 [Thecamonas trahens ATCC 50062] |
| c79675.graph_c0 | 9.75E-04 | 0.7183 | up | uncharacterized protein LOC108912051 [Anoplophora glabripennis] |
| c84333.graph_c0 | 6.56E-05 | 0.7161 | up | putative sugar transporter 5 [Phaedon cochleariae] |
| c87308.graph_c0 | 5.15E-04 | 0.7148 | up | None |
| c81395.graph_c0 | 9.53E-03 | 0.7125 | up | 72 kDa inositol polyphosphate 5-phosphatase [Leptinotarsa decemlineata] |
| c76655.graph_c0 | 5.55E-03 | 0.7113 | up | myb-like protein X [Leptinotarsa decemlineata] |
| c76845.graph_c0 | 1.18E-04 | 0.7047 | up | None |
| c69944.graph_c0 | 9.89E-03 | 0.7024 | up | None |
| c69581.graph_c0 | 3.57E-03 | 0.7009 | up | alkaline phosphatase [Anoplophora glabripennis] |
| c66329.graph_c0 | 4.99E-04 | 0.6938 | up | tetra-peptide repeat homeobox protein 1-like [Anoplophora glabripennis] |
| c77682.graph_c0 | 2.58E-03 | 0.6893 | up | None |
| c87626.graph_c0 | 1.54E-04 | 0.6885 | up | None |
| c85283.graph_c0 | 3.45E-03 | 0.6877 | up | None |
| c77063.graph_c1 | 1.19E-03 | 0.6838 | up | UMP-CMP kinase 2, mitochondrial-like [Leptinotarsa decemlineata] |
| c83012.graph_c0 | 8.66E-03 | 0.6805 | up | lysozyme precursor [Tribolium castaneum] |
| c87227.graph_c0 | 7.92E-03 | 0.6728 | up | None |
| c74840.graph_c0 | 7.87E-03 | 0.6718 | up | None |
| c67628.graph_c0 | 7.98E-03 | 0.6715 | up | protein tyrosine phosphatase type IVA 1 isoform X1 [Leptinotarsa decemlineata] |
| c38077.graph_c0 | 1.00E-03 | 0.6713 | up | uncharacterized protein LOC111514894 [Leptinotarsa decemlineata] |
| c73323.graph_c0 | 5.66E-03 | 0.6647 | up | beta-lactamase-like protein 2 homolog [Anoplophora glabripennis] |
| c89242.graph_c0 | 9.32E-03 | 0.6619 | up | pyrroline-5-carboxylate synthetase [Leptinotarsa decemlineata] |
| c85827.graph_c0 | 3.02E-03 | 0.6603 | up | PREDICTED: uncharacterized protein LOC753896 [Strongylocentrotus purpuratus] |
| c69020.graph_c0 | 5.89E-05 | 0.6600 | up | uncharacterized protein LOC108914446 [Anoplophora glabripennis] |
| c73944.graph_c0 | 3.92E-03 | 0.6599 | up | Niemann-Pick type protein homolog 1B [Anoplophora glabripennis] |
| c78297.graph_c0 | 1.50E-03 | 0.6576 | up | tenascin-like [Leptinotarsa decemlineata] |
| c54438.graph_c0 | 1.55E-04 | 0.6558 | up | hypothetical protein YQE_03480, partial [Dendroctonus ponderosae] |
| c86349.graph_c0 | 6.53E-05 | 0.6553 | up | protein javelin [Leptinotarsa decemlineata] |
| c71278.graph_c0 | 2.77E-07 | 0.6483 | up | uncharacterized protein LOC108913225 [Anoplophora glabripennis] |
| c85328.graph_c0 | 4.94E-03 | 0.6412 | up | leucine-rich repeat-containing G-protein coupled receptor 4 isoform X1 [Leptinotarsa decemlineata] |
| c80303.graph_c0 | 1.84E-03 | 0.6405 | up | None |
| c89258.graph_c0 | 3.37E-03 | 0.6400 | up | hypothetical protein B7P43_G06602 [Cryptotermes secundus] |
| c89031.graph_c0 | 1.88E-08 | 0.6398 | up | hypothetical protein ALC62_05268 [Cyphomyrmex costatus] |
| c47152.graph_c0 | 2.26E-04 | 0.6398 | up | None |
| c88570.graph_c0 | 5.61E-03 | 0.6384 | up | None |
| c77648.graph_c0 | 1.04E-06 | 0.6349 | up | None |
| c86486.graph_c0 | 1.16E-05 | 0.6347 | up | PREDICTED: uncharacterized protein LOC100142506 isoform X1 [Tribolium castaneum] |
| c68188.graph_c0 | 6.78E-03 | 0.6314 | up | beta-galactosidase-1-like protein 2 [Anoplophora glabripennis] |
| c61277.graph_c0 | 1.91E-03 | 0.6266 | up | hypothetical protein YQE_00075, partial [Dendroctonus ponderosae] |
| c69086.graph_c0 | 8.92E-03 | 0.6258 | up | None |
| c83400.graph_c0 | 7.71E-04 | 0.6252 | up | None |
| c81878.graph_c0 | 1.16E-03 | 0.6245 | up | nose resistant to fluoxetine protein 6-like isoform X1 [Anoplophora glabripennis] |
| c89554.graph_c0 | 5.15E-04 | 0.6239 | up | None |
| c78852.graph_c0 | 1.75E-05 | 0.6111 | up | uncharacterized protein LOC111502128 isoform X1 [Leptinotarsa decemlineata] |
| c83711.graph_c0 | 1.25E-04 | 0.6096 | up | uncharacterized protein LOC111516889 [Leptinotarsa decemlineata] |
| c81661.graph_c0 | 1.18E-03 | 0.6086 | up | uncharacterized protein LOC105842971 [Bombyx mori] |
| c76405.graph_c0 | 3.53E-03 | 0.5995 | up | fatty acyl-CoA reductase wat-like [Anoplophora glabripennis] |
| c88086.graph_c0 | 2.50E-04 | 0.5979 | up | transposable element tc3 transposase [Lasius niger] |
| c87246.graph_c0 | 2.13E-03 | 0.5976 | up | adenylate cyclase type 6 isoform X2 [Anoplophora glabripennis] |
| c82606.graph_c1 | 7.94E-03 | 0.5937 | up | None |
| c84407.graph_c0 | 4.07E-03 | 0.5928 | up | ATP-binding cassette transporter [Chrysomela tremula] |
| c87740.graph_c0 | 2.42E-04 | 0.5918 | up | glycerol-3-phosphate dehydrogenase, mitochondrial isoform X1 [Leptinotarsa decemlineata] |
| c70895.graph_c0 | 6.88E-06 | 0.5913 | up | leucine-rich repeat protein SHOC-2-like isoform X1 [Leptinotarsa decemlineata] |
| c74796.graph_c0 | 2.50E-06 | 0.5890 | up | peroxidasin, partial [Anoplophora glabripennis] |
| c62332.graph_c0 | 1.60E-05 | -0.5860 | down | troponin C, isotype gamma-like [Anoplophora glabripennis] |
| c86227.graph_c0 | 1.72E-06 | -0.5911 | down | endoribonuclease Dicer-like [Leptinotarsa decemlineata] |
| c78716.graph_c0 | 1.66E-03 | -0.5952 | down | homogentisate 1,2-dioxygenase [Leptinotarsa decemlineata] |
| c74727.graph_c0 | 4.10E-03 | -0.6001 | down | cilia- and flagella-associated protein 58-like [Leptinotarsa decemlineata] |
| c83974.graph_c0 | 4.00E-03 | -0.6006 | down | trifunctional purine biosynthetic protein adenosine-3 [Aethina tumida] |
| c88050.graph_c0 | 4.53E-03 | -0.6041 | down | PiggyBac transposable element-derived protein 4, partial [Stegodyphus mimosarum] |
| c81677.graph_c0 | 1.65E-04 | -0.6048 | down | protein msta-like [Leptinotarsa decemlineata] |
| c86693.graph_c0 | 5.39E-03 | -0.6102 | down | L-lactate dehydrogenase isoform X2 [Leptinotarsa decemlineata] |
| c47071.graph_c0 | 5.27E-03 | -0.6107 | down | PREDICTED: crustapain-like [Dendroctonus ponderosae] |
| c83651.graph_c0 | 3.30E-03 | -0.6127 | down | inter-alpha-trypsin inhibitor heavy chain H4-like isoform X8 [Leptinotarsa decemlineata] |
| c79123.graph_c0 | 2.34E-03 | -0.6146 | down | uncharacterized protein LOC111505890 [Leptinotarsa decemlineata] |
| c86266.graph_c0 | 8.92E-03 | -0.6299 | down | hypothetical protein EAG_05435 [Camponotus floridanus] |
| c80136.graph_c0 | 6.83E-05 | -0.6308 | down | putative glycosyl hydrolase [Chrysomela lapponica] |
| c76013.graph_c0 | 3.52E-04 | -0.6317 | down | mitochondrial basic amino acids transporter-like [Leptinotarsa decemlineata] |
| c84729.graph_c0 | 9.67E-03 | -0.6443 | down | uncharacterized protein LOC108905377 [Anoplophora glabripennis] |
| c78532.graph_c0 | 4.38E-06 | -0.6454 | down | PREDICTED: selenium-binding protein 1 [Tribolium castaneum] |
| c81089.graph_c0 | 1.04E-03 | -0.6461 | down | putative helicase mov-10-B.1 [Leptinotarsa decemlineata] |
| c74955.graph_c0 | 1.54E-08 | -0.6471 | down | hypothetical protein YQE_06088, partial [Dendroctonus ponderosae] |
| c82409.graph_c0 | 5.02E-03 | -0.6524 | down | beta-galactosidase-1-like protein 3 [Leptinotarsa decemlineata] |
| c83201.graph_c0 | 8.87E-03 | -0.6530 | down | zinc finger protein 423-like isoform X1 [Leptinotarsa decemlineata] |
| c76386.graph_c0 | 7.83E-05 | -0.6632 | down | juvenile hormone esterase-like [Leptinotarsa decemlineata] |
| c84358.graph_c1 | 1.69E-04 | -0.6673 | down | jerky protein homolog-like [Ceratitis capitata] |
| c80122.graph_c1 | 1.26E-04 | -0.6687 | down | ornithine aminotransferase, mitochondrial [Anoplophora glabripennis] |
| c64556.graph_c1 | 6.65E-03 | -0.6705 | down | chemosensory protein [Galeruca daurica] |
| c60507.graph_c0 | 8.50E-04 | -0.6731 | down | uncharacterized protein LOC111514586 [Leptinotarsa decemlineata] |
| c81882.graph_c0 | 1.53E-04 | -0.6792 | down | multidrug resistance-associated protein 4-like [Leptinotarsa decemlineata] |
| c64028.graph_c0 | 3.20E-04 | -0.6830 | down | odorant-binding protein [Galeruca daurica] |
| c88458.graph_c0 | 8.86E-03 | -0.6888 | down | heat shock protein 68-like [Leptinotarsa decemlineata] |
| c85166.graph_c0 | 2.39E-04 | -0.6891 | down | tudor domain-containing protein 7 isoform X2 [Anoplophora glabripennis] |
| c85088.graph_c0 | 2.52E-03 | -0.6910 | down | PREDICTED: coiled-coil domain-containing protein AGAP005037 isoform X10 [Tribolium castaneum] |
| c70919.graph_c0 | 7.02E-06 | -0.6928 | down | uncharacterized protein LOC111504342 [Leptinotarsa decemlineata] |
| c69550.graph_c0 | 5.57E-04 | -0.6930 | down | None |
| c74889.graph_c0 | 3.78E-05 | -0.6985 | down | FGGY carbohydrate kinase domain-containing protein [Anoplophora glabripennis] |
| c80269.graph_c0 | 7.60E-03 | -0.7068 | down | peroxidasin-like protein [Anoplophora glabripennis] |
| c77741.graph_c0 | 9.05E-03 | -0.7094 | down | uncharacterized protein LOC111510432 [Leptinotarsa decemlineata] |
| c85339.graph_c0 | 1.53E-05 | -0.7125 | down | None |
| c83319.graph_c3 | 1.32E-04 | -0.7174 | down | balbiani ring protein 3-like [Anoplophora glabripennis] |
| c64060.graph_c0 | 4.68E-09 | -0.7194 | down | probable chitinase 10 [Anoplophora glabripennis] |
| c84433.graph_c0 | 5.56E-03 | -0.7237 | down | PREDICTED: uncharacterized protein LOC103522310 [Diaphorina citri] |
| c78947.graph_c0 | 2.30E-06 | -0.7277 | down | glycerol-3-phosphate dehydrogenase [NAD(+)], cytoplasmic [Leptinotarsa decemlineata] |
| c87284.graph_c0 | 1.50E-05 | -0.7308 | down | fatty acid synthase 2, partial [Colaphellus bowringi] |
| c81648.graph_c0 | 1.43E-05 | -0.7331 | down | regucalcin-like isoform X2 [Leptinotarsa decemlineata] |
| c74156.graph_c0 | 8.80E-03 | -0.7487 | down | UDP-glucuronosyltransferase 2B33-like [Anoplophora glabripennis] |
| c86899.graph_c0 | 8.56E-03 | -0.7494 | down | uncharacterized protein LOC111419767 [Onthophagus taurus] |
| c71827.graph_c0 | 3.21E-05 | -0.7523 | down | purine nucleoside phosphorylase isoform X3 [Leptinotarsa decemlineata] |
| c66793.graph_c0 | 9.98E-03 | -0.7534 | down | uncharacterized protein LOC108912127 [Anoplophora glabripennis] |
| c79283.graph_c0 | 1.71E-04 | -0.7595 | down | glycoside hydrolase family 1 [Phyllotreta striolata] |
| c78686.graph_c0 | 4.59E-04 | -0.7618 | down | PREDICTED: LOW QUALITY PROTEIN: aspartate aminotransferase, mitochondrial [Aethina tumida] |
| c73029.graph_c0 | 1.95E-03 | -0.7735 | down | tubulin polyglutamylase TTLL4 isoform X1 [Anoplophora glabripennis] |
| c81248.graph_c0 | 2.09E-06 | -0.7760 | down | PAN2-PAN3 deadenylation complex catalytic subunit PAN2 [Anoplophora glabripennis] |
| c89559.graph_c0 | 3.99E-04 | -0.7793 | down | PREDICTED: zinc finger BED domain-containing protein 1-like [Diuraphis noxia] |
| c86485.graph_c0 | 9.27E-03 | -0.7804 | down | mucin-5AC-like [Anoplophora glabripennis] |
| c79494.graph_c0 | 7.10E-05 | -0.7805 | down | fumarylacetoacetate hydrolase domain-containing protein 2-like [Leptinotarsa decemlineata] |
| c75643.graph_c0 | 3.56E-09 | -0.7819 | down | lysophospholipid acyltransferase 1 [Leptinotarsa decemlineata] |
| c77897.graph_c1 | 6.21E-04 | -0.7865 | down | None |
| c77863.graph_c0 | 4.53E-03 | -0.7944 | down | peritrophin-1-like [Anoplophora glabripennis] |
| c88421.graph_c0 | 5.41E-03 | -0.7991 | down | tigger transposable element-derived protein 6-like, partial [Myzus persicae] |
| c76850.graph_c0 | 6.79E-04 | -0.8068 | down | esterase [Leptinotarsa decemlineata] |
| c81372.graph_c0 | 8.96E-03 | -0.8096 | down | uncharacterized protein LOC111362437 [Spodoptera litura] |
| c87215.graph_c0 | 9.51E-04 | -0.8097 | down | None |
| c86297.graph_c0 | 7.67E-09 | -0.8182 | down | nidogen-2 [Anoplophora glabripennis] |
| c86608.graph_c0 | 8.33E-12 | -0.8201 | down | heterogeneous nuclear ribonucleoprotein Q isoform X2 [Anoplophora glabripennis] |
| c39060.graph_c0 | 2.95E-08 | -0.8208 | down | None |
| c88877.graph_c0 | 2.60E-06 | -0.8234 | down | glucose dehydrogenase [FAD, quinone]-like [Leptinotarsa decemlineata] |
| c70709.graph_c0 | 7.67E-03 | -0.8244 | down | BICD family-like cargo adapter 1 [Leptinotarsa decemlineata] |
| c79395.graph_c0 | 8.03E-03 | -0.8247 | down | PREDICTED: piggyBac transposable element-derived protein 4-like [Amyelois transitella] |
| c69603.graph_c0 | 1.40E-03 | -0.8275 | down | alpha-tocopherol transfer protein-like [Anoplophora glabripennis] |
| c82416.graph_c0 | 1.29E-03 | -0.8287 | down | uncharacterized protein LOC108905167 [Anoplophora glabripennis] |
| c77136.graph_c0 | 5.31E-03 | -0.8333 | down | sodium leak channel non-selective protein isoform X2 [Anoplophora glabripennis] |
| c85899.graph_c0 | 4.30E-03 | -0.8350 | down | PREDICTED: uncharacterized protein LOC106053576 [Biomphalaria glabrata] |
| c72988.graph_c0 | 8.63E-07 | -0.8465 | down | protein obstructor-E-like [Leptinotarsa decemlineata] |
| c83806.graph_c0 | 4.41E-06 | -0.8501 | down | amidophosphoribosyltransferase-like [Leptinotarsa decemlineata] |
| c66945.graph_c0 | 3.76E-03 | -0.8516 | down | None |
| c69135.graph_c0 | 1.45E-03 | -0.8769 | down | glycine-rich RNA-binding protein 8 [Leptinotarsa decemlineata] |
| c75649.graph_c0 | 2.41E-07 | -0.8774 | down | PREDICTED: lambda-crystallin [Tribolium castaneum] |
| c79880.graph_c0 | 1.61E-03 | -0.8788 | down | facilitated trehalose transporter Tret1-like isoform X1 [Leptinotarsa decemlineata] |
| c63566.graph_c0 | 3.03E-03 | -0.8836 | down | PREDICTED: uncharacterized protein LOC109601824 [Aethina tumida] |
| c58730.graph_c1 | 2.79E-05 | -0.8936 | down | peptidoglycan-recognition protein-SC2 [Tenebrio molitor] |
| c84083.graph_c0 | 4.55E-04 | -0.8991 | down | myogenesis-regulating glycosidase-like [Anoplophora glabripennis] |
| c89996.graph_c1 | 2.47E-03 | -0.9139 | down | -- |
| c82256.graph_c0 | 1.49E-12 | -0.9166 | down | None |
| c58730.graph_c0 | 2.63E-03 | -0.9186 | down | PREDICTED: peptidoglycan-recognition protein SC2 [Tribolium castaneum] |
| c87611.graph_c0 | 2.69E-03 | -0.9328 | down | PREDICTED: adenylate kinase 9 [Dendroctonus ponderosae] |
| c80850.graph_c0 | 6.37E-04 | -0.9458 | down | radical S-adenosyl methionine domain-containing protein 2-like [Leptinotarsa decemlineata] |
| c86639.graph_c0 | 4.11E-06 | -0.9471 | down | ecdysone-induced protein 74EF isoform A [Leptinotarsa decemlineata] |
| c88563.graph_c0 | 2.88E-06 | -0.9760 | down | uncharacterized protein LOC111504487 [Leptinotarsa decemlineata] |
| c87632.graph_c0 | 2.46E-10 | -1.0057 | down | heterogeneous nuclear ribonucleoprotein Q [Leptinotarsa decemlineata] |
| c69643.graph_c0 | 5.69E-05 | -1.0066 | down | retinoid-inducible serine carboxypeptidase-like [Leptinotarsa decemlineata] |
| c83674.graph_c0 | 1.83E-15 | -1.0220 | down | piggyBac transposable element-derived protein 3-like [Leptinotarsa decemlineata] |
| c78626.graph_c0 | 9.52E-04 | -1.0283 | down | bromodomain-containing protein DDB_G0280777 [Anoplophora glabripennis] |
| c37561.graph_c0 | 2.48E-09 | -1.0336 | down | PREDICTED: crustapain-like [Dendroctonus ponderosae] |
| c78473.graph_c0 | 7.66E-04 | -1.0454 | down | transposase [Pectinophora gossypiella] |
| c67871.graph_c0 | 4.71E-07 | -1.0610 | down | glycine N-methyltransferase [Anoplophora glabripennis] |
| c64926.graph_c0 | 3.10E-05 | -1.0649 | down | None |
| c79880.graph_c1 | 4.52E-04 | -1.0855 | down | facilitated trehalose transporter Tret1 [Anoplophora glabripennis] |
| c85895.graph_c0 | 1.59E-12 | -1.1050 | down | luciferin 4-monooxygenase-like [Leptinotarsa decemlineata] |
| c81243.graph_c0 | 2.83E-04 | -1.1132 | down | None |
| c81799.graph_c0 | 1.21E-04 | -1.1324 | down | None |
| c84589.graph_c0 | 2.23E-07 | -1.1600 | down | protein msta-like [Leptinotarsa decemlineata] |
| c83026.graph_c0 | 3.82E-07 | -1.1928 | down | PREDICTED: RNA-directed DNA polymerase from mobile element jockey-like [Bemisia tabaci] |
| c70155.graph_c0 | 4.38E-05 | -1.2576 | down | None |
| c71232.graph_c0 | 2.92E-21 | -1.2699 | down | aldose 1-epimerase-like [Leptinotarsa decemlineata] |
| c72112.graph_c0 | 3.84E-08 | -1.3599 | down | facilitated trehalose transporter Tret1-like [Leptinotarsa decemlineata] |
| c73343.graph_c0 | 1.15E-05 | -1.3647 | down | None |
| c82886.graph_c0 | 4.97E-09 | -1.4007 | down | nose resistant to fluoxetine protein 6-like isoform X1 [Anoplophora glabripennis] |
| c82942.graph_c0 | 9.87E-09 | -1.4122 | down | PREDICTED: uncharacterized protein LOC109541399 [Dendroctonus ponderosae] |
| c67727.graph_c0 | 8.63E-07 | -1.5313 | down | None |
| CKb vs Tb | | | | |
| c75346.graph_c0 | 2.23E-07 | 2.0023 | up | -- |
| c57561.graph_c0 | 8.07E-07 | 1.8823 | up | None |
| c76431.graph_c0 | 1.28E-06 | 1.8691 | up | None |
| c71279.graph_c0 | 2.22E-05 | 1.6339 | up | PREDICTED: F-box/WD repeat-containing protein 7-like [Papilio xuthus] |
| c90339.graph_c0 | 3.17E-05 | 1.6029 | up | PREDICTED: probable proteasome subunit beta type-4 [Papilio xuthus] |
| c39641.graph_c0 | 3.29E-05 | 1.6005 | up | PREDICTED: ATP-binding cassette sub-family G member 1-like [Papilio xuthus] |
| c50641.graph_c0 | 3.59E-05 | 1.5914 | up | PREDICTED: uncharacterized protein C24B11.05-like [Papilio xuthus] |
| c82101.graph_c3 | 2.06E-06 | 1.5853 | up | odorant-binding protein 5 [Pyrrhalta maculicollis] |
| c75443.graph_c0 | 4.39E-05 | 1.5792 | up | -- |
| c74827.graph_c0 | 4.39E-05 | 1.5577 | up | uncharacterized protein LOC111513065 [Leptinotarsa decemlineata] |
| c65352.graph_c0 | 6.23E-05 | 1.5381 | up | PREDICTED: V-type proton ATPase subunit c&apos;&apos;2-like [Papilio xuthus] |
| c55864.graph_c0 | 8.84E-05 | 1.5071 | up | PREDICTED: ATP-binding cassette sub-family G member 1-like [Papilio xuthus] |
| c72104.graph_c0 | 1.08E-04 | 1.4903 | up | -- |
| c72629.graph_c0 | 1.17E-04 | 1.4895 | up | PREDICTED: transcription initiation factor TFIID subunit 5-like [Papilio xuthus] |
| c55287.graph_c0 | 1.37E-04 | 1.4709 | up | None |
| c71025.graph_c0 | 1.65E-04 | 1.4511 | up | PREDICTED: ABC transporter B family member 5-like [Papilio xuthus] |
| c71460.graph_c0 | 1.74E-04 | 1.4336 | up | antichymotrypsin-2-like isoform X2 [Leptinotarsa decemlineata] |
| c69765.graph_c0 | 2.06E-04 | 1.4331 | up | Cytoskeleton-associated protein domain containing protein, partial [Haemonchus contortus] |
| c71227.graph_c0 | 2.19E-04 | 1.4273 | up | PREDICTED: uncharacterized protein LOC106120438 [Papilio xuthus] |
| c73498.graph_c0 | 2.21E-04 | 1.4103 | up | None |
| c76875.graph_c0 | 2.61E-04 | 1.4073 | up | PREDICTED: NEDD8 [Drosophila ficusphila] |
| c90129.graph_c0 | 2.81E-04 | 1.4040 | up | 40S ribosomal protein S17 [Tetrahymena thermophila SB210] |
| c75791.graph_c0 | 2.81E-04 | 1.4033 | up | None |
| c39229.graph_c0 | 3.04E-04 | 1.3961 | up | PREDICTED: GPN-loop GTPase 3-like [Aplysia californica] |
| c71328.graph_c1 | 2.68E-04 | 1.3822 | up | None |
| c82613.graph_c0 | 5.96E-06 | 1.3799 | up | fatty acid synthase-like [Leptinotarsa decemlineata] |
| c25973.graph_c0 | 3.53E-04 | 1.3722 | up | -- |
| c59654.graph_c0 | 4.93E-04 | 1.3467 | up | PREDICTED: eukaryotic translation initiation factor 6-like [Papilio xuthus] |
| c74277.graph_c0 | 4.98E-04 | 1.3462 | up | PREDICTED: probable DNA repair helicase RAD25 homolog [Papilio xuthus] |
| c68953.graph_c0 | 4.85E-04 | 1.3408 | up | None |
| c71050.graph_c0 | 5.45E-04 | 1.3360 | up | None |
| c19186.graph_c0 | 5.61E-04 | 1.3194 | up | PREDICTED: ubiquitin-conjugating enzyme E2 15-like [Papilio xuthus] |
| c80524.graph_c0 | 6.52E-04 | 1.3137 | up | PREDICTED: exportin-1-like [Papilio xuthus] |
| c75939.graph_c0 | 6.34E-04 | 1.3105 | up | None |
| c79483.graph_c0 | 5.94E-04 | 1.3072 | up | PREDICTED: cytosolic Fe-S cluster assembly factor NUBP2 homolog [Papilio xuthus] |
| c85279.graph_c0 | 3.53E-04 | 1.3032 | up | vitellogenin-like [Leptinotarsa decemlineata] |
| c56748.graph_c0 | 6.66E-04 | 1.3021 | up | dehydrogenase/reductase SDR family member 13-like [Leptinotarsa decemlineata] |
| c71589.graph_c0 | 6.56E-04 | 1.2981 | up | None |
| c71188.graph_c0 | 7.13E-04 | 1.2981 | up | None |
| c39857.graph_c0 | 8.39E-04 | 1.2893 | up | None |
| c82351.graph_c0 | 1.48E-04 | 1.2860 | up | None |
| c65158.graph_c0 | 7.13E-04 | 1.2807 | up | None |
| c69965.graph_c0 | 7.44E-04 | 1.2801 | up | None |
| c62068.graph_c0 | 7.88E-04 | 1.2738 | up | PREDICTED: deoxyhypusine synthase-like [Papilio xuthus] |
| c70796.graph_c0 | 8.98E-04 | 1.2701 | up | carboxy-terminal kinesin 2-like [Exaiptasia pallida] |
| c66039.graph_c0 | 7.72E-04 | 1.2699 | up | PREDICTED: EKC/KEOPS complex subunit BUD32-like [Papilio xuthus] |
| c74996.graph_c0 | 9.23E-04 | 1.2669 | up | -- |
| c66641.graph_c0 | 9.52E-04 | 1.2603 | up | PREDICTED: uncharacterized protein LOC106118770, partial [Papilio xuthus] |
| c70952.graph_c0 | 9.88E-04 | 1.2582 | up | None |
| c19194.graph_c0 | 1.14E-03 | 1.2554 | up | None |
| c64476.graph_c0 | 1.16E-03 | 1.2518 | up | PREDICTED: serine/threonine-protein phosphatase 2A activator-like [Papilio xuthus] |
| c68831.graph_c0 | 6.78E-04 | 1.2490 | up | PREDICTED: uncharacterized protein LOC108741931 [Agrilus planipennis] |
| c73524.graph_c0 | 1.16E-03 | 1.2488 | up | PREDICTED: forkhead box protein A2-A-like [Acropora digitifera] |
| c64059.graph_c0 | 1.21E-03 | 1.2477 | up | LP13955p [Drosophila melanogaster] |
| c65889.graph_c0 | 1.17E-03 | 1.2465 | up | PREDICTED: probable E3 ubiquitin-protein ligase MARCH10 [Ciona intestinalis] |
| c69774.graph_c0 | 1.13E-03 | 1.2299 | up | PREDICTED: LOW QUALITY PROTEIN: transcription initiation factor IIE subunit alpha-like [Papilio xuthus] |
| c47753.graph_c0 | 1.24E-03 | 1.2297 | up | PREDICTED: DNA-directed RNA polymerase II subunit rpb7-like [Polistes dominula] |
| c71191.graph_c0 | 3.35E-05 | 1.2275 | up | probable pseudouridine-5&apos;-phosphatase [Leptinotarsa decemlineata] |
| c67706.graph_c0 | 1.23E-03 | 1.2245 | up | None |
| c37734.graph_c0 | 1.50E-03 | 1.2245 | up | -- |
| c73395.graph_c0 | 1.34E-03 | 1.2238 | up | PREDICTED: uncharacterized protein LOC106114956 [Papilio xuthus] |
| c73728.graph_c0 | 1.34E-03 | 1.2195 | up | -- |
| c78929.graph_c0 | 1.45E-03 | 1.2177 | up | PREDICTED: probable cell cycle serine/threonine-protein kinase CDC5 homolog [Papilio xuthus] |
| c75038.graph_c0 | 1.47E-03 | 1.2127 | up | PREDICTED: ribonuclease H2 subunit A-like [Papilio xuthus] |
| c40366.graph_c0 | 1.35E-03 | 1.2102 | up | None |
| c77911.graph_c0 | 1.51E-03 | 1.2093 | up | None |
| c77848.graph_c0 | 1.54E-03 | 1.2088 | up | PREDICTED: probable serine/threonine-protein kinase RIO1 homolog, partial [Papilio xuthus] |
| c55086.graph_c0 | 1.54E-03 | 1.2037 | up | PREDICTED: NADH-cytochrome b5 reductase 2-like [Papilio xuthus] |
| c48442.graph_c0 | 1.78E-03 | 1.2007 | up | None |
| c72968.graph_c0 | 1.72E-03 | 1.1963 | up | hypothetical protein TRIADDRAFT_57795 [Trichoplax adhaerens] |
| c67390.graph_c0 | 1.94E-03 | 1.1910 | up | hypothetical protein PBRA_001981 [Plasmodiophora brassicae] |
| c64633.graph_c0 | 2.04E-03 | 1.1909 | up | PREDICTED: probable cell division control protein 7 homolog 1 [Papilio xuthus] |
| c74206.graph_c0 | 1.65E-03 | 1.1888 | up | None |
| c24826.graph_c0 | 1.66E-03 | 1.1838 | up | -- |
| c39726.graph_c0 | 1.78E-03 | 1.1791 | up | PREDICTED: eukaryotic translation initiation factor 2-alpha kinase 2-like [Papilio xuthus] |
| c72941.graph_c0 | 1.62E-03 | 1.1762 | up | PREDICTED: cell division cycle protein 16 homolog [Branchiostoma belcheri] |
| c57035.graph_c0 | 2.12E-03 | 1.1749 | up | None |
| c71269.graph_c0 | 2.40E-03 | 1.1732 | up | Solute carrier family 2, facilitated glucose transporter member 5 [Schistosoma japonicum] |
| c70071.graph_c0 | 2.41E-03 | 1.1693 | up | proteasome Regulatory Particle, Non-ATPase-like [Caenorhabditis elegans] |
| c77042.graph_c0 | 2.39E-03 | 1.1685 | up | zinc finger protein 585A [Anoplophora glabripennis] |
| c53911.graph_c0 | 2.61E-03 | 1.1630 | up | None |
| c61891.graph_c0 | 2.41E-03 | 1.1591 | up | None |
| c75787.graph_c0 | 2.16E-03 | 1.1588 | up | PREDICTED: probable replication factor C subunit 3 [Papilio xuthus] |
| c74291.graph_c0 | 2.59E-03 | 1.1562 | up | -- |
| c19482.graph_c0 | 2.65E-03 | 1.1560 | up | None |
| c67069.graph_c0 | 2.23E-03 | 1.1558 | up | None |
| c86895.graph_c0 | 8.53E-04 | 1.1532 | up | cilia- and flagella-associated protein 47-like [Leptinotarsa decemlineata] |
| c52696.graph_c0 | 2.48E-03 | 1.1497 | up | None |
| c73566.graph_c0 | 2.47E-03 | 1.1471 | up | PREDICTED: serine/threonine-protein kinase MRCK alpha-like [Papilio xuthus] |
| c61584.graph_c0 | 2.35E-03 | 1.1465 | up | PREDICTED: general transcription factor IIF subunit 2-like [Papilio xuthus] |
| c67671.graph_c0 | 2.45E-03 | 1.1457 | up | None |
| c66434.graph_c1 | 2.55E-03 | 1.1435 | up | PREDICTED: E3 ubiquitin-protein ligase At1g63170-like [Papilio xuthus] |
| c80598.graph_c0 | 3.04E-03 | 1.1417 | up | piggyBac transposable element-derived protein 4-like [Myzus persicae] |
| c68407.graph_c0 | 2.87E-06 | 1.1414 | up | uncharacterized protein LOC111504345 [Leptinotarsa decemlineata] |
| c69115.graph_c0 | 2.91E-03 | 1.1379 | up | PREDICTED: probable heat shock factor protein homolog [Papilio xuthus] |
| c65537.graph_c0 | 2.70E-03 | 1.1367 | up | PREDICTED: putative subtilisin-like proteinase 2 [Papilio xuthus] |
| c88372.graph_c0 | 2.50E-04 | 1.1264 | up | fatty acid synthase-like [Leptinotarsa decemlineata] |
| c65250.graph_c0 | 3.28E-03 | 1.1235 | up | taf9 RNA polymerase tata box binding protein -associated isoform cra_b, putative [Ichthyophthirius multifiliis] |
| c60439.graph_c0 | 3.22E-03 | 1.1135 | up | -- |
| c55010.graph_c0 | 3.30E-03 | 1.1117 | up | PREDICTED: 60S ribosome subunit biogenesis protein NIP7 homolog [Papilio xuthus] |
| c87527.graph_c0 | 3.72E-03 | 1.1068 | up | PREDICTED: uncharacterized protein LOC105555964 [Vollenhovia emeryi] |
| c83495.graph_c0 | 2.51E-03 | 1.1030 | up | hypothetical protein BOX15_Mlig002331g1 [Macrostomum lignano] |
| c39745.graph_c0 | 2.13E-03 | 1.1015 | up | None |
| c85272.graph_c1 | 3.51E-03 | 1.0964 | up | PREDICTED: Fanconi anemia group J protein homolog [Ceratosolen solmsi marchali] |
| c65469.graph_c0 | 3.75E-03 | 1.0936 | up | PREDICTED: facilitated trehalose transporter Tret1 isoform X1 [Drosophila takahashii] |
| c70433.graph_c0 | 1.95E-06 | 1.0931 | up | growth/differentiation factor 8-like [Leptinotarsa decemlineata] |
| c48503.graph_c0 | 3.85E-03 | 1.0928 | up | -- |
| c67713.graph_c0 | 4.17E-03 | 1.0894 | up | PREDICTED: probable CTD kinase subunit alpha homolog [Papilio xuthus] |
| c63973.graph_c0 | 4.21E-03 | 1.0838 | up | None |
| c67457.graph_c0 | 3.44E-03 | 1.0826 | up | MAPK/MAK/MRK overlapping kinase-like, partial [Anoplophora glabripennis] |
| c73089.graph_c0 | 4.11E-03 | 1.0810 | up | PREDICTED: probable magnesium transporter NIPA8 [Papilio xuthus] |
| c70225.graph_c0 | 4.87E-03 | 1.0809 | up | PREDICTED: diphthine--ammonia ligase-like [Papilio xuthus] |
| c70266.graph_c0 | 4.62E-03 | 1.0755 | up | PREDICTED: bromodomain testis-specific protein-like [Papilio xuthus] |
| c76895.graph_c0 | 5.05E-03 | 1.0750 | up | None |
| c61712.graph_c0 | 4.55E-03 | 1.0725 | up | PREDICTED: transcription factor TFIIIB component B&apos;&apos;-like [Papilio xuthus] |
| c61494.graph_c0 | 4.64E-03 | 1.0696 | up | PREDICTED: anaphase-promoting complex subunit cdc20-like [Papilio xuthus] |
| c65899.graph_c0 | 5.34E-03 | 1.0683 | up | hypothetical protein H696_02079 [Fonticula alba] |
| c50656.graph_c0 | 4.78E-03 | 1.0683 | up | None |
| c64156.graph_c0 | 4.06E-03 | 1.0636 | up | None |
| c63742.graph_c0 | 5.65E-03 | 1.0571 | up | PREDICTED: eukaryotic translation initiation factor 3 subunit I-like [Papilio xuthus] |
| c72364.graph_c0 | 5.21E-03 | 1.0569 | up | PREDICTED: uncharacterized protein LOC106114853 [Papilio xuthus] |
| c77996.graph_c0 | 5.43E-03 | 1.0561 | up | Replication factor C subunit 1 [Thelohanellus kitauei] |
| c37527.graph_c0 | 6.39E-03 | 1.0532 | up | hypothetical protein SAMD00019534_012180 [Acytostelium subglobosum LB1] |
| c66021.graph_c0 | 5.94E-03 | 1.0516 | up | None |
| c70986.graph_c0 | 5.84E-03 | 1.0508 | up | PREDICTED: myosin heavy chain kinase B-like [Papilio xuthus] |
| c25528.graph_c0 | 5.34E-03 | 1.0496 | up | PREDICTED: GTP-binding protein ryh1-like [Papilio xuthus] |
| c69115.graph_c1 | 5.33E-03 | 1.0485 | up | PREDICTED: acyl-CoA thioesterase 2-like [Papilio machaon] |
| c66547.graph_c0 | 6.10E-03 | 1.0460 | up | PREDICTED: phenylalanine--tRNA ligase alpha subunit isoform X2 [Fopius arisanus] |
| c50623.graph_c0 | 5.34E-03 | 1.0455 | up | PREDICTED: CDP-diacylglycerol--inositol 3-phosphatidyltransferase-like [Hydra vulgaris] |
| c90258.graph_c0 | 5.94E-03 | 1.0454 | up | PREDICTED: transcription initiation factor TFIID subunit 11-like [Papilio xuthus] |
| c71602.graph_c0 | 5.16E-03 | 1.0452 | up | None |
| c54408.graph_c0 | 5.95E-03 | 1.0429 | up | PREDICTED: mRNA cap guanine-N7 methyltransferase-like [Papilio xuthus] |
| c75134.graph_c0 | 6.77E-03 | 1.0419 | up | PREDICTED: fidgetin-like protein 1 isoform X3 [Drosophila kikkawai] |
| c72403.graph_c0 | 6.96E-03 | 1.0385 | up | uncharacterized protein LOC105842288 [Bombyx mori] |
| c80710.graph_c0 | 2.07E-03 | 1.0372 | up | beta-glucuronidase-like protein [Leptinotarsa decemlineata] |
| c67553.graph_c0 | 6.08E-03 | 1.0295 | up | Chondroitin sulfate proteoglycan 4 [Trichinella pseudospiralis] |
| c69591.graph_c0 | 6.05E-03 | 1.0277 | up | PREDICTED: monoacylglycerol lipase ABHD12-like [Saccoglossus kowalevskii] |
| c66467.graph_c0 | 6.26E-03 | 1.0248 | up | None |
| c73600.graph_c0 | 7.15E-03 | 1.0248 | up | -- |
| c79839.graph_c0 | 7.10E-03 | 1.0248 | up | PREDICTED: LOW QUALITY PROTEIN: uncharacterized protein LOC108664000 [Hyalella azteca] |
| c79206.graph_c0 | 6.55E-03 | 1.0243 | up | hypothetical protein TRIADDRAFT_57642 [Trichoplax adhaerens] |
| c64382.graph_c0 | 6.82E-03 | 1.0236 | up | PREDICTED: sporulation-specific protein 15-like, partial [Hyalella azteca] |
| c65339.graph_c0 | 7.04E-03 | 1.0214 | up | PREDICTED: uncharacterized protein LOC109545082 [Dendroctonus ponderosae] |
| c61645.graph_c0 | 6.95E-03 | 1.0196 | up | phosphatidylinositol transfer protein beta isoform-like [Stylophora pistillata] |
| c70752.graph_c0 | 5.55E-03 | 1.0186 | up | PREDICTED: uncharacterized protein LOC106127983 [Papilio xuthus] |
| c63989.graph_c0 | 6.62E-03 | 1.0083 | up | PREDICTED: LOW QUALITY PROTEIN: DNA-directed RNA polymerase II subunit RPB3-like, partial [Papilio xuthus] |
| c38778.graph_c0 | 7.60E-03 | 1.0053 | up | PREDICTED: nuclear cap-binding protein subunit 2-like [Papilio xuthus] |
| c85874.graph_c0 | 3.41E-03 | 0.9989 | up | trichohyalin isoform X2 [Leptinotarsa decemlineata] |
| c49895.graph_c0 | 7.28E-03 | 0.9970 | up | PREDICTED: pre-mRNA 3&apos;-end-processing factor FIP1 [Galendromus occidentalis] |
| c61755.graph_c0 | 8.13E-03 | 0.9948 | up | ADP-ribosylation factor GTPase-a, putative (macronuclear) [Oxytricha trifallax] |
| c71296.graph_c0 | 9.29E-03 | 0.9921 | up | PREDICTED: probable serine/threonine-protein kinase mkcD [Papilio xuthus] |
| c83155.graph_c0 | 6.62E-03 | 0.9902 | up | uncharacterized protein LOC111359630 [Spodoptera litura] |
| c19015.graph_c0 | 8.28E-03 | 0.9883 | up | uncharacterized protein LOC6646184 isoform X3 [Drosophila willistoni] |
| c40061.graph_c0 | 5.83E-04 | 0.9877 | up | mitotic spindle assembly checkpoint protein MAD2A [Leptinotarsa decemlineata] |
| c70743.graph_c0 | 7.94E-03 | 0.9833 | up | -- |
| c73765.graph_c0 | 8.13E-03 | 0.9814 | up | None |
| c75970.graph_c0 | 9.01E-03 | 0.9799 | up | hypothetical protein DDB_G0279451 [Dictyostelium discoideum AX4] |
| c74404.graph_c0 | 8.80E-03 | 0.9778 | up | PREDICTED: probable kinetochore protein NUF2 [Papilio xuthus] |
| c61597.graph_c0 | 9.73E-03 | 0.9773 | up | fatty acid elongase [Thecamonas trahens ATCC 50062] |
| c61484.graph_c0 | 8.45E-03 | 0.9761 | up | None |
| c39642.graph_c0 | 9.20E-03 | 0.9755 | up | None |
| c70165.graph_c0 | 8.75E-03 | 0.9721 | up | PREDICTED: cleavage stimulation factor subunit 3-like [Hydra vulgaris] |
| c69728.graph_c0 | 9.19E-03 | 0.9711 | up | None |
| c68456.graph_c0 | 9.14E-03 | 0.9694 | up | -- |
| c66165.graph_c0 | 9.95E-03 | 0.9688 | up | PREDICTED: solute carrier family 25 member 38-A-like isoform X2 [Nicrophorus vespilloides] |
| c74811.graph_c0 | 6.65E-03 | 0.9666 | up | PREDICTED: cilia- and flagella-associated protein 53-like [Dendroctonus ponderosae] |
| c74253.graph_c1 | 9.47E-03 | 0.9662 | up | None |
| c71045.graph_c0 | 8.70E-03 | 0.9660 | up | hypothetical protein RvY_15626 [Ramazzottius varieornatus] |
| c65745.graph_c0 | 9.46E-03 | 0.9587 | up | -- |
| c64457.graph_c0 | 9.10E-03 | 0.9505 | up | PREDICTED: replication factor C subunit 4-like [Papilio xuthus] |
| c75977.graph_c0 | 3.58E-03 | 0.9413 | up | cell division cycle-associated protein 3-like isoform X2 [Leptinotarsa decemlineata] |
| c68915.graph_c0 | 9.77E-03 | 0.9412 | up | PREDICTED: eukaryotic peptide chain release factor subunit 1-like [Papilio xuthus] |
| c80699.graph_c0 | 4.77E-04 | 0.9334 | up | uncharacterized protein LOC111055686 [Nilaparvata lugens] |
| c65594.graph_c0 | 3.49E-04 | 0.9283 | up | elongation of very long chain fatty acids protein AAEL008004 isoform X4 [Ceratitis capitata] |
| c81353.graph_c1 | 1.44E-04 | 0.9236 | up | GDP-fucose protein O-fucosyltransferase 1 [Leptinotarsa decemlineata] |
| c81878.graph_c0 | 1.02E-03 | 0.9115 | up | nose resistant to fluoxetine protein 6-like isoform X1 [Anoplophora glabripennis] |
| c63743.graph_c0 | 6.91E-03 | 0.9046 | up | None |
| c85647.graph_c0 | 9.96E-04 | 0.9031 | up | PREDICTED: uncharacterized protein LOC103309185 [Acyrthosiphon pisum] |
| c59836.graph_c0 | 8.44E-03 | 0.9024 | up | aquaporin-like isoform X1 [Leptinotarsa decemlineata] |
| c74243.graph_c0 | 1.75E-03 | 0.8982 | up | pancreatic lipase-related protein 2-like [Leptinotarsa decemlineata] |
| c71082.graph_c0 | 4.50E-04 | 0.8885 | up | tektin-3-like [Leptinotarsa decemlineata] |
| c75733.graph_c0 | 5.52E-09 | 0.8767 | up | farnesol dehydrogenase-like [Leptinotarsa decemlineata] |
| c73083.graph_c0 | 8.54E-03 | 0.8720 | up | lymphokine-activated killer T-cell-originated protein kinase homolog [Leptinotarsa decemlineata] |
| c80223.graph_c0 | 3.99E-03 | 0.8566 | up | Krueppel homolog 1-like isoform X1 [Anoplophora glabripennis] |
| c81985.graph_c0 | 4.30E-03 | 0.8286 | up | intraflagellar transport protein 80 homolog [Anoplophora glabripennis] |
| c73192.graph_c0 | 7.44E-04 | 0.8274 | up | protein yellow [Leptinotarsa decemlineata] |
| c69581.graph_c0 | 1.49E-03 | 0.8227 | up | alkaline phosphatase [Anoplophora glabripennis] |
| c61844.graph_c0 | 8.59E-04 | 0.8128 | up | small heat shock protein Hsp20.6 [Galeruca daurica] |
| c66284.graph_c0 | 6.57E-04 | 0.8109 | up | ribonuclease P protein subunit p29 [Anoplophora glabripennis] |
| c80249.graph_c0 | 1.29E-03 | 0.8077 | up | None |
| c79041.graph_c0 | 8.80E-03 | 0.8057 | up | THO complex subunit 7 homolog [Leptinotarsa decemlineata] |
| c60846.graph_c0 | 1.00E-02 | 0.8038 | up | None |
| c87602.graph_c0 | 2.65E-04 | 0.7992 | up | citron Rho-interacting kinase [Anoplophora glabripennis] |
| c86852.graph_c0 | 7.18E-03 | 0.7957 | up | coiled-coil domain-containing protein 40 [Leptinotarsa decemlineata] |
| c89602.graph_c0 | 1.38E-03 | 0.7924 | up | male-specific doublesex isoform m isoform 2 [Tribolium castaneum] |
| c68438.graph_c0 | 7.20E-03 | 0.7825 | up | lysosomal Pro-X carboxypeptidase-like [Anoplophora glabripennis] |
| c82939.graph_c3 | 4.74E-04 | 0.7813 | up | PREDICTED: uncharacterized protein LOC105669381 isoform X1 [Linepithema humile] |
| c71706.graph_c0 | 1.04E-04 | 0.7807 | up | None |
| c50681.graph_c0 | 8.13E-03 | 0.7701 | up | carboxypeptidase N subunit 2-like [Centruroides sculpturatus] |
| c83030.graph_c0 | 2.88E-03 | 0.7697 | up | uncharacterized protein LOC111504873 [Leptinotarsa decemlineata] |
| c81609.graph_c0 | 5.54E-04 | 0.7571 | up | None |
| c86432.graph_c0 | 1.42E-03 | 0.7405 | up | multidrug resistance-associated protein 4 [Anoplophora glabripennis] |
| c85171.graph_c0 | 4.44E-03 | 0.7353 | up | uncharacterized protein LOC111502827 [Leptinotarsa decemlineata] |
| c85130.graph_c0 | 5.71E-04 | 0.7352 | up | cyclin-dependent kinase 1 [Leptinotarsa decemlineata] |
| c80964.graph_c0 | 3.14E-03 | 0.7225 | up | uncharacterized protein LOC108917702 [Anoplophora glabripennis] |
| c69493.graph_c0 | 4.51E-03 | 0.7216 | up | PREDICTED: uncharacterized protein LOC109541831 [Dendroctonus ponderosae] |
| c55388.graph_c0 | 2.94E-03 | 0.7180 | up | protein brambleberry [Anoplophora glabripennis] |
| c86588.graph_c0 | 5.10E-03 | 0.7112 | up | None |
| c62194.graph_c0 | 7.14E-03 | 0.6997 | up | None |
| c83700.graph_c0 | 2.82E-03 | 0.6967 | up | serine/threonine-protein kinase polo [Anoplophora glabripennis] |
| c88304.graph_c0 | 1.13E-03 | 0.6961 | up | PREDICTED: uncharacterized protein LOC105556050 [Vollenhovia emeryi] |
| c84407.graph_c0 | 6.81E-04 | 0.6831 | up | ATP-binding cassette transporter [Chrysomela tremula] |
| c78529.graph_c0 | 3.10E-03 | 0.6783 | up | uncharacterized protein LOC111509433 isoform X1 [Leptinotarsa decemlineata] |
| c87601.graph_c0 | 4.86E-04 | 0.6771 | up | None |
| c85832.graph_c0 | 8.23E-03 | 0.6762 | up | copia protein, partial [Lasius niger] |
| c78019.graph_c0 | 1.95E-03 | 0.6715 | up | cytochrome c [Zootermopsis nevadensis] |
| c88219.graph_c0 | 1.98E-03 | 0.6668 | up | uncharacterized protein LOC110996927 isoform X1 [Pieris rapae] |
| c68284.graph_c0 | 9.71E-03 | 0.6624 | up | G2/mitotic-specific cyclin-B2-like isoform X1 [Leptinotarsa decemlineata] |
| c78368.graph_c0 | 2.36E-04 | 0.6624 | up | serine protease 7-like [Anoplophora glabripennis] |
| c87476.graph_c0 | 4.44E-03 | 0.6566 | up | TOG array regulator of axonemal microtubules protein 2-like isoform X1 [Anoplophora glabripennis] |
| c77158.graph_c0 | 7.64E-03 | 0.6563 | up | methionyl-tRNA formyltransferase, mitochondrial [Leptinotarsa decemlineata] |
| c75948.graph_c0 | 4.26E-03 | 0.6559 | up | asparagine synthetase domain-containing protein CG17486 [Anoplophora glabripennis] |
| c64098.graph_c0 | 1.92E-03 | 0.6501 | up | hypothetical protein TcasGA2_TC032144 [Tribolium castaneum] |
| c86167.graph_c0 | 8.91E-05 | 0.6478 | up | spondin-1 [Leptinotarsa decemlineata] |
| c78297.graph_c0 | 3.13E-03 | 0.6473 | up | tenascin-like [Leptinotarsa decemlineata] |
| c82898.graph_c0 | 2.89E-03 | 0.6381 | up | disks large-associated protein 5-like [Leptinotarsa decemlineata] |
| c75995.graph_c0 | 2.48E-03 | 0.6374 | up | None |
| c38485.graph_c0 | 4.20E-05 | 0.6343 | up | retinol-binding protein pinta [Anoplophora glabripennis] |
| c77595.graph_c0 | 4.99E-07 | 0.6249 | up | protein patched homolog 1 isoform X1 [Anoplophora glabripennis] |
| c81659.graph_c0 | 1.17E-04 | 0.6247 | up | multidrug resistance-associated protein [Phaedon cochleariae] |
| c79820.graph_c1 | 1.97E-03 | 0.6183 | up | DNA-directed RNA polymerase III subunit RPC2 [Anoplophora glabripennis] |
| c75067.graph_c0 | 9.43E-03 | 0.6174 | up | 4-coumarate--CoA ligase-like [Leptinotarsa decemlineata] |
| c80095.graph_c0 | 5.67E-03 | 0.6166 | up | maltase 2-like isoform X2 [Leptinotarsa decemlineata] |
| c86514.graph_c0 | 9.14E-03 | 0.6157 | up | None |
| c88308.graph_c0 | 2.82E-03 | 0.6143 | up | uncharacterized protein LOC105841937 [Bombyx mori] |
| c87738.graph_c0 | 9.72E-03 | 0.6071 | up | uncharacterized protein LOC111513503 [Leptinotarsa decemlineata] |
| c88437.graph_c0 | 3.54E-03 | 0.6011 | up | synaptotagmin 1 isoform X2 [Leptinotarsa decemlineata] |
| c58927.graph_c0 | 1.59E-03 | 0.5898 | up | mitochondrial import inner membrane translocase subunit Tim22 [Leptinotarsa decemlineata] |
| c71092.graph_c0 | 2.85E-03 | 0.5864 | up | ribonuclease H2 subunit A [Anoplophora glabripennis] |
| c39991.graph_c0 | 1.85E-04 | 0.5853 | up | PREDICTED: deoxynucleotidyltransferase terminal-interacting protein 2 [Tribolium castaneum] |
| c83327.graph_c0 | 5.88E-06 | -0.5863 | down | None |
| c68068.graph_c0 | 3.68E-03 | -0.5870 | down | None |
| c77653.graph_c1 | 5.51E-03 | -0.5893 | down | glucose dehydrogenase [FAD, quinone]-like isoform X1 [Leptinotarsa decemlineata] |
| c80268.graph_c0 | 8.80E-03 | -0.5897 | down | PREDICTED: LOW QUALITY PROTEIN: flocculation protein FLO11-like [Aethina tumida] |
| c69778.graph_c0 | 3.79E-03 | -0.5919 | down | putative juvenile hormone esterase 2 [Colaphellus bowringi] |
| c64808.graph_c1 | 9.16E-03 | -0.5923 | down | glycoside hydrolase family 1 [Phyllotreta striolata] |
| c75166.graph_c0 | 9.83E-03 | -0.5927 | down | RecName: Full=Phenoloxidase-activating factor 2; AltName: Full=45 KDa PPAF; Short=Hd-45; AltName: Full=Prophenoloxidase-activating factor II; AltName: Full=Serine protease-like PPAF-2; Contains: RecName: Full=Phenoloxidase-activating factor 2 light chain; Contains: RecName: Full=Phenoloxidase-activating factor 2 heavy chain; Flags: Precursor |
| c81171.graph_c0 | 5.40E-04 | -0.5929 | down | venom carboxylesterase-6-like [Leptinotarsa decemlineata] |
| c65260.graph_c0 | 3.84E-04 | -0.5951 | down | caspase-like protein [Tribolium castaneum] |
| c79806.graph_c0 | 2.61E-03 | -0.5953 | down | putative glutathione S-transferase epsilon class member 7 [Leptinotarsa decemlineata] |
| c24853.graph_c0 | 8.45E-04 | -0.5957 | down | None |
| c76163.graph_c0 | 2.03E-03 | -0.5991 | down | alpha-N-acetylgalactosaminidase [Anoplophora glabripennis] |
| c67709.graph_c0 | 2.05E-03 | -0.6034 | down | uncharacterized protein LOC111502474 [Leptinotarsa decemlineata] |
| c64866.graph_c0 | 3.89E-03 | -0.6036 | down | putative antimicrobial peptide acaloleptin A, partial [Phaedon cochleariae] |
| c75752.graph_c1 | 5.99E-08 | -0.6038 | down | None |
| c88998.graph_c0 | 4.85E-03 | -0.6049 | down | None |
| c89525.graph_c0 | 6.57E-04 | -0.6052 | down | uncharacterized protein LOC111047292 [Nilaparvata lugens] |
| c76707.graph_c0 | 3.65E-07 | -0.6058 | down | angiotensin-converting enzyme-like [Leptinotarsa decemlineata] |
| c85486.graph_c0 | 6.59E-04 | -0.6071 | down | choline transporter-like protein 1 [Anoplophora glabripennis] |
| c81317.graph_c0 | 6.96E-04 | -0.6118 | down | alpha-mannosidase 2 [Anoplophora glabripennis] |
| c81565.graph_c0 | 9.99E-03 | -0.6127 | down | nose resistant to fluoxetine protein 6-like isoform X1 [Anoplophora glabripennis] |
| c89486.graph_c0 | 8.43E-03 | -0.6144 | down | Transposable element Tc3 transposase-like Protein [Tribolium castaneum] |
| c85229.graph_c0 | 5.26E-03 | -0.6150 | down | uncharacterized protein LOC111514347 [Leptinotarsa decemlineata] |
| c80931.graph_c0 | 6.13E-03 | -0.6152 | down | insecticide resistance-associated cytochrome P450 [Diabrotica virgifera virgifera] |
| c69650.graph_c1 | 1.04E-08 | -0.6166 | down | stress-associated endoplasmic reticulum protein 2 isoform X2 [Anoplophora glabripennis] |
| c49538.graph_c0 | 7.51E-03 | -0.6167 | down | uncharacterized protein LOC111511581 isoform X1 [Leptinotarsa decemlineata] |
| c75001.graph_c0 | 1.95E-03 | -0.6169 | down | UDP-glucuronosyltransferase 2B7-like [Anoplophora glabripennis] |
| c74469.graph_c0 | 2.87E-05 | -0.6171 | down | juvenile hormone acid O-methyltransferase-like [Leptinotarsa decemlineata] |
| c87041.graph_c0 | 4.94E-03 | -0.6196 | down | uncharacterized protein LOC108905016 [Anoplophora glabripennis] |
| c87215.graph_c0 | 9.42E-03 | -0.6202 | down | None |
| c72988.graph_c0 | 6.38E-03 | -0.6205 | down | protein obstructor-E-like [Leptinotarsa decemlineata] |
| c77343.graph_c0 | 1.64E-04 | -0.6213 | down | esterase [Leptinotarsa decemlineata] |
| c66115.graph_c0 | 2.76E-06 | -0.6221 | down | glutathione S-transferase 1-like [Leptinotarsa decemlineata] |
| c80924.graph_c0 | 3.03E-04 | -0.6240 | down | alanine--glyoxylate aminotransferase 2-like [Leptinotarsa decemlineata] |
| c69607.graph_c0 | 4.31E-03 | -0.6249 | down | dipeptidyl peptidase [Leptinotarsa decemlineata] |
| c87742.graph_c0 | 4.76E-03 | -0.6252 | down | None |
| c84990.graph_c0 | 2.38E-06 | -0.6255 | down | None |
| c70116.graph_c0 | 3.19E-03 | -0.6257 | down | protein takeout-like [Leptinotarsa decemlineata] |
| c81211.graph_c1 | 9.65E-03 | -0.6282 | down | uncharacterized protein LOC111418272 [Onthophagus taurus] |
| c87134.graph_c1 | 9.28E-04 | -0.6296 | down | la-related protein 6 [Leptinotarsa decemlineata] |
| c83415.graph_c0 | 1.40E-04 | -0.6334 | down | neprilysin-1 [Anoplophora glabripennis] |
| c85837.graph_c0 | 1.25E-03 | -0.6340 | down | fructose-1,6-bisphosphatase 1 isoform X2 [Anoplophora glabripennis] |
| c79189.graph_c0 | 8.08E-03 | -0.6347 | down | 1,2-dihydroxy-3-keto-5-methylthiopentene dioxygenase [Anoplophora glabripennis] |
| c65822.graph_c0 | 4.59E-03 | -0.6374 | down | putative acyl-CoA-binding protein [Leptinotarsa decemlineata] |
| c86364.graph_c0 | 1.04E-03 | -0.6378 | down | lysosomal alpha-mannosidase [Anoplophora glabripennis] |
| c81677.graph_c0 | 8.07E-05 | -0.6387 | down | protein msta-like [Leptinotarsa decemlineata] |
| c78342.graph_c0 | 4.37E-04 | -0.6395 | down | glycoside hydrolase family 1 [Phyllotreta striolata] |
| c70470.graph_c0 | 5.08E-05 | -0.6411 | down | None |
| c78123.graph_c0 | 3.01E-03 | -0.6415 | down | von Willebrand factor D and EGF domain-containing protein-like [Leptinotarsa decemlineata] |
| c69715.graph_c0 | 7.04E-03 | -0.6415 | down | chorion class B protein L12-like [Leptinotarsa decemlineata] |
| c82891.graph_c1 | 3.72E-03 | -0.6434 | down | complement C1r subcomponent-like [Leptinotarsa decemlineata] |
| c81679.graph_c0 | 7.71E-03 | -0.6448 | down | glutathione hydrolase 7-like [Anoplophora glabripennis] |
| c86016.graph_c1 | 5.20E-04 | -0.6460 | down | unknown [Dendroctonus ponderosae] |
| c74734.graph_c0 | 1.05E-05 | -0.6489 | down | uncharacterized protein LOC111517989 [Leptinotarsa decemlineata] |
| c82859.graph_c0 | 3.91E-03 | -0.6496 | down | leucine-rich repeat-containing protein 15-like [Leptinotarsa decemlineata] |
| c87284.graph_c0 | 5.15E-03 | -0.6505 | down | fatty acid synthase 2, partial [Colaphellus bowringi] |
| c81349.graph_c0 | 1.19E-06 | -0.6511 | down | uncharacterized protein LOC111502600 [Leptinotarsa decemlineata] |
| c85763.graph_c0 | 1.29E-05 | -0.6512 | down | glycoside hydrolase family 45 protein [Phaedon cochleariae] |
| c70495.graph_c0 | 1.82E-03 | -0.6542 | down | juvenile hormone epoxide hydrolase 1-like isoform X2 [Anoplophora glabripennis] |
| c89263.graph_c0 | 1.91E-04 | -0.6545 | down | hypothetical protein X975_10844, partial [Stegodyphus mimosarum] |
| c69084.graph_c0 | 9.18E-03 | -0.6556 | down | odorant-binding protein 11, partial [Pyrrhalta aenescens] |
| c86103.graph_c0 | 6.70E-03 | -0.6563 | down | anoctamin-5 isoform X1 [Anoplophora glabripennis] |
| c81882.graph_c0 | 5.37E-03 | -0.6607 | down | multidrug resistance-associated protein 4-like [Leptinotarsa decemlineata] |
| c48860.graph_c0 | 6.86E-04 | -0.6636 | down | putative juvenile hormone esterase 2 [Colaphellus bowringi] |
| c68312.graph_c0 | 6.80E-04 | -0.6675 | down | None |
| c71208.graph_c0 | 8.68E-04 | -0.6678 | down | pickpocket protein 28-like [Leptinotarsa decemlineata] |
| c79963.graph_c1 | 3.45E-04 | -0.6713 | down | major facilitator superfamily domain-containing protein 6 [Anoplophora glabripennis] |
| c24466.graph_c0 | 1.46E-03 | -0.6718 | down | protein CTLA-2-alpha-like isoform X1 [Anoplophora glabripennis] |
| c64934.graph_c0 | 6.43E-03 | -0.6759 | down | uncharacterized protein LOC111502473 [Leptinotarsa decemlineata] |
| c87169.graph_c0 | 4.54E-03 | -0.6765 | down | PREDICTED: LOW QUALITY PROTEIN: neural-cadherin-like [Aethina tumida] |
| c77575.graph_c0 | 1.14E-03 | -0.6770 | down | glycoside hydrolase family 1 [Phyllotreta striolata] |
| c76535.graph_c0 | 1.45E-03 | -0.6793 | down | mucin-5AC-like [Leptinotarsa decemlineata] |
| c62332.graph_c0 | 1.23E-04 | -0.6822 | down | troponin C, isotype gamma-like [Anoplophora glabripennis] |
| c79738.graph_c0 | 8.10E-03 | -0.6838 | down | GTPase-activating protein [Anoplophora glabripennis] |
| c80058.graph_c0 | 5.06E-04 | -0.6840 | down | arylsulfatase B [Anoplophora glabripennis] |
| c50571.graph_c0 | 9.01E-03 | -0.6906 | down | None |
| c78503.graph_c0 | 2.52E-04 | -0.6916 | down | beta-galactosidase-1-like protein 2 [Anoplophora glabripennis] |
| c80940.graph_c0 | 8.43E-03 | -0.6940 | down | glutathione S-transferase 1-like [Leptinotarsa decemlineata] |
| c73756.graph_c0 | 1.18E-03 | -0.6965 | down | -- |
| c66273.graph_c0 | 4.15E-03 | -0.6965 | down | 60S ribosomal protein L5 [Anoplophora glabripennis] |
| c86638.graph_c0 | 1.55E-03 | -0.6976 | down | endocuticle structural glycoprotein SgAbd-4-like [Leptinotarsa decemlineata] |
| c83518.graph_c0 | 4.29E-03 | -0.6986 | down | glutathione hydrolase 1 proenzyme-like [Leptinotarsa decemlineata] |
| c74163.graph_c0 | 1.39E-04 | -0.7003 | down | None |
| c88996.graph_c0 | 3.22E-05 | -0.7027 | down | glycoside hydrolase family 1 [Phyllotreta striolata] |
| c83745.graph_c0 | 4.41E-03 | -0.7043 | down | bumetanide-sensitive sodium-(potassium)-chloride cotransporter [Leptinotarsa decemlineata] |
| c89956.graph_c0 | 1.79E-03 | -0.7074 | down | myb-like protein P [Leptinotarsa decemlineata] |
| c81646.graph_c0 | 1.78E-03 | -0.7079 | down | uncharacterized protein LOC111515837 [Leptinotarsa decemlineata] |
| c88577.graph_c0 | 1.83E-07 | -0.7115 | down | PREDICTED: high affinity copper uptake protein 1 isoform X2 [Dendroctonus ponderosae] |
| c83016.graph_c0 | 4.48E-03 | -0.7129 | down | PREDICTED: aldehyde dehydrogenase, mitochondrial [Tribolium castaneum] |
| c73773.graph_c0 | 4.27E-05 | -0.7130 | down | 5-formyltetrahydrofolate cyclo-ligase [Leptinotarsa decemlineata] |
| c79436.graph_c0 | 8.32E-03 | -0.7135 | down | uncharacterized protein LOC111510100 [Leptinotarsa decemlineata] |
| c70717.graph_c1 | 1.68E-04 | -0.7167 | down | putative juvenile hormone esterase 2 [Colaphellus bowringi] |
| c69046.graph_c0 | 3.62E-03 | -0.7187 | down | uncharacterized protein LOC111503465 [Leptinotarsa decemlineata] |
| c81748.graph_c0 | 4.01E-04 | -0.7194 | down | scavenger receptor class B member 1-like isoform X1 [Anoplophora glabripennis] |
| c85839.graph_c0 | 3.07E-03 | -0.7195 | down | None |
| c85740.graph_c0 | 6.33E-05 | -0.7201 | down | glycoside hydrolase family 28 [Diabrotica virgifera virgifera] |
| c73885.graph_c3 | 1.90E-04 | -0.7228 | down | glycoside hydrolase family 1 [Phyllotreta striolata] |
| c74856.graph_c0 | 3.44E-06 | -0.7246 | down | None |
| c66596.graph_c0 | 3.65E-03 | -0.7252 | down | uncharacterized protein LOC111512244 [Leptinotarsa decemlineata] |
| c74029.graph_c0 | 4.39E-05 | -0.7271 | down | neutral alpha-glucosidase C isoform X2 [Anoplophora glabripennis] |
| c79269.graph_c0 | 1.17E-04 | -0.7284 | down | ankyrin-3-like [Pseudomyrmex gracilis] |
| c88904.graph_c0 | 1.76E-03 | -0.7288 | down | uncharacterized protein LOC108910939 [Anoplophora glabripennis] |
| c73176.graph_c0 | 9.95E-03 | -0.7327 | down | uncharacterized protein LOC111052559 [Nilaparvata lugens] |
| c64023.graph_c0 | 9.29E-03 | -0.7345 | down | odorant-binding protein [Galeruca daurica] |
| c81407.graph_c0 | 6.26E-09 | -0.7350 | down | None |
| c60024.graph_c0 | 6.46E-04 | -0.7364 | down | uncharacterized protein LOC111502601 [Leptinotarsa decemlineata] |
| c57241.graph_c0 | 1.04E-06 | -0.7397 | down | perlucin-like [Anoplophora glabripennis] |
| c89473.graph_c0 | 8.97E-03 | -0.7401 | down | kelch-like protein 17 [Anoplophora glabripennis] |
| c84480.graph_c0 | 6.51E-03 | -0.7416 | down | uncharacterized protein LOC111504847 [Leptinotarsa decemlineata] |
| c66285.graph_c0 | 1.36E-07 | -0.7425 | down | PREDICTED: uncharacterized protein LOC109598355 [Aethina tumida] |
| c82405.graph_c0 | 3.04E-03 | -0.7443 | down | prostatic acid phosphatase-like isoform X1 [Anoplophora glabripennis] |
| c57163.graph_c0 | 4.11E-08 | -0.7447 | down | uncharacterized protein LOC108910120 isoform X1 [Anoplophora glabripennis] |
| c69951.graph_c0 | 4.15E-03 | -0.7449 | down | uncharacterized protein LOC111511067 [Leptinotarsa decemlineata] |
| c67974.graph_c0 | 1.10E-04 | -0.7531 | down | C1 family cathepsin L11 [Tenebrio molitor] |
| c62267.graph_c0 | 1.03E-04 | -0.7538 | down | 3-hydroxyisobutyrate dehydrogenase, mitochondrial [Leptinotarsa decemlineata] |
| c82510.graph_c1 | 2.18E-03 | -0.7541 | down | chitinase-like protein Idgf4 [Leptinotarsa decemlineata] |
| c89138.graph_c0 | 2.06E-04 | -0.7549 | down | hypothetical protein B5V51_9240 [Heliothis virescens] |
| c56043.graph_c0 | 6.52E-03 | -0.7567 | down | None |
| c72849.graph_c0 | 3.71E-03 | -0.7612 | down | uncharacterized protein LOC108905821 isoform X2 [Anoplophora glabripennis] |
| c80621.graph_c0 | 9.30E-04 | -0.7620 | down | uncharacterized protein LOC108907575 [Anoplophora glabripennis] |
| c74370.graph_c0 | 7.60E-03 | -0.7625 | down | leucine-rich repeat-containing protein 15-like [Leptinotarsa decemlineata] |
| c77979.graph_c2 | 6.19E-07 | -0.7638 | down | GILT-like protein 1 [Leptinotarsa decemlineata] |
| c50451.graph_c0 | 6.92E-04 | -0.7639 | down | lipopolysaccharide-induced tumor necrosis factor-alpha factor homolog [Leptinotarsa decemlineata] |
| c62736.graph_c0 | 8.14E-06 | -0.7640 | down | esterase [Leptinotarsa decemlineata] |
| c77037.graph_c0 | 1.93E-05 | -0.7644 | down | active phase-associated protein II [Gastrophysa atrocyanea] |
| c87202.graph_c0 | 8.82E-03 | -0.7649 | down | uncharacterized protein LOC108904526 isoform X1 [Anoplophora glabripennis] |
| c86071.graph_c0 | 1.33E-04 | -0.7662 | down | D-aspartate oxidase [Leptinotarsa decemlineata] |
| c74837.graph_c0 | 2.29E-03 | -0.7674 | down | protein yellow-like [Leptinotarsa decemlineata] |
| c78390.graph_c0 | 7.68E-03 | -0.7707 | down | UPF0439 protein C9orf30-like protein, partial [Harpegnathos saltator] |
| c77035.graph_c0 | 8.85E-03 | -0.7732 | down | None |
| c65296.graph_c0 | 3.09E-06 | -0.7750 | down | protein CTLA-2-alpha-like isoform X3 [Anoplophora glabripennis] |
| c64752.graph_c0 | 1.88E-04 | -0.7786 | down | spidroin-1-like [Leptinotarsa decemlineata] |
| c66113.graph_c0 | 1.91E-05 | -0.7804 | down | None |
| c85218.graph_c0 | 2.76E-03 | -0.7811 | down | uncharacterized protein LOC111515749, partial [Leptinotarsa decemlineata] |
| c81168.graph_c1 | 1.51E-04 | -0.7863 | down | glycoside hydrolase family 1 [Phyllotreta striolata] |
| c88344.graph_c0 | 5.02E-04 | -0.7865 | down | PREDICTED: uncharacterized protein LOC103314057 [Tribolium castaneum] |
| c39647.graph_c0 | 1.65E-08 | -0.7884 | down | None |
| c83979.graph_c0 | 1.76E-08 | -0.7886 | down | sorting nexin-16 [Leptinotarsa decemlineata] |
| c70346.graph_c0 | 6.51E-03 | -0.7910 | down | uncharacterized protein LOC111425111 [Onthophagus taurus] |
| c82972.graph_c0 | 5.72E-04 | -0.7917 | down | uncharacterized protein LOC106672751 [Cimex lectularius] |
| c62003.graph_c0 | 4.89E-03 | -0.7930 | down | None |
| c86360.graph_c0 | 9.20E-03 | -0.7930 | down | None |
| c82204.graph_c0 | 6.60E-03 | -0.7932 | down | PREDICTED: uncharacterized protein LOC107073003 [Polistes dominula] |
| c69617.graph_c0 | 8.70E-05 | -0.7953 | down | PREDICTED: uncharacterized protein LOC103312905 [Tribolium castaneum] |
| c82988.graph_c0 | 3.92E-03 | -0.7982 | down | putative glutathione S-transferase zeta class member 1 [Leptinotarsa decemlineata] |
| c82349.graph_c0 | 3.25E-03 | -0.7988 | down | juvenile hormone esterase-like [Leptinotarsa decemlineata] |
| c19425.graph_c0 | 8.97E-03 | -0.7993 | down | None |
| c70717.graph_c0 | 7.09E-05 | -0.8001 | down | venom carboxylesterase-6-like isoform X1 [Leptinotarsa decemlineata] |
| c62298.graph_c0 | 6.54E-03 | -0.8014 | down | putative inorganic phosphate cotransporter [Anoplophora glabripennis] |
| c70943.graph_c0 | 1.93E-03 | -0.8023 | down | uncharacterized protein LOC108908189 [Anoplophora glabripennis] |
| c24787.graph_c0 | 1.49E-04 | -0.8040 | down | hypothetical protein YQE_08855, partial [Dendroctonus ponderosae] |
| c70449.graph_c0 | 3.41E-04 | -0.8082 | down | ferritin, heavy subunit [Anoplophora glabripennis] |
| c48538.graph_c0 | 1.62E-07 | -0.8084 | down | uncharacterized protein LOC107442817 [Parasteatoda tepidariorum] |
| c64485.graph_c0 | 5.01E-03 | -0.8086 | down | flexible cuticle protein 12-like [Leptinotarsa decemlineata] |
| c75189.graph_c0 | 5.15E-04 | -0.8091 | down | None |
| c78739.graph_c1 | 5.83E-03 | -0.8115 | down | None |
| c69550.graph_c0 | 3.00E-04 | -0.8115 | down | None |
| c73058.graph_c0 | 3.72E-08 | -0.8117 | down | beta-1,3-glucan-binding protein-like [Leptinotarsa decemlineata] |
| c85064.graph_c1 | 5.58E-03 | -0.8120 | down | None |
| c87366.graph_c0 | 3.47E-03 | -0.8125 | down | RNA-directed DNA polymerase from mobile element jockey [Cryptotermes secundus] |
| c81489.graph_c0 | 8.54E-04 | -0.8151 | down | uncharacterized protein LOC111692495 [Anoplophora glabripennis] |
| c75781.graph_c0 | 2.31E-04 | -0.8156 | down | GILT-like protein 1 isoform X2 [Anoplophora glabripennis] |
| c77609.graph_c0 | 1.58E-04 | -0.8158 | down | putative glutathione S-transferase delta class member 3 [Leptinotarsa decemlineata] |
| c71016.graph_c0 | 8.01E-03 | -0.8191 | down | PREDICTED: LOW QUALITY PROTEIN: microtubule-associated proteins 1A/1B light chain 3C-like [Aethina tumida] |
| c67757.graph_c0 | 2.18E-04 | -0.8215 | down | glycoside hydrolase family 28 [Diabrotica virgifera virgifera] |
| c81457.graph_c2 | 1.33E-07 | -0.8218 | down | putative cystathionine gamma-lyase 2 [Leptinotarsa decemlineata] |
| c77178.graph_c0 | 1.19E-07 | -0.8221 | down | antichymotrypsin-2-like [Leptinotarsa decemlineata] |
| c78401.graph_c1 | 1.26E-03 | -0.8269 | down | myrosinase 1 [Anoplophora glabripennis] |
| c78947.graph_c0 | 6.15E-04 | -0.8272 | down | glycerol-3-phosphate dehydrogenase [NAD(+)], cytoplasmic [Leptinotarsa decemlineata] |
| c69672.graph_c0 | 1.28E-06 | -0.8332 | down | synaptic vesicle membrane protein VAT-1 homolog-like [Leptinotarsa decemlineata] |
| c74232.graph_c0 | 3.46E-03 | -0.8334 | down | asialoglycoprotein receptor 2 [Leptinotarsa decemlineata] |
| c74414.graph_c0 | 4.52E-03 | -0.8371 | down | actin cytoskeleton-regulatory complex protein PAN1-like [Leptinotarsa decemlineata] |
| c49001.graph_c0 | 3.00E-03 | -0.8404 | down | protein spaetzle 5 [Anoplophora glabripennis] |
| c88202.graph_c0 | 3.47E-03 | -0.8423 | down | uncharacterized protein LOC111049714 [Nilaparvata lugens] |
| c80550.graph_c0 | 3.31E-07 | -0.8431 | down | beta-mannosidase [Anoplophora glabripennis] |
| c83152.graph_c0 | 1.82E-03 | -0.8433 | down | esterase [Leptinotarsa decemlineata] |
| c69631.graph_c0 | 1.42E-03 | -0.8439 | down | endocuticle structural glycoprotein SgAbd-4-like [Anoplophora glabripennis] |
| c37451.graph_c0 | 2.47E-03 | -0.8446 | down | odorant-binding protein 11, partial [Pyrrhalta aenescens] |
| c88916.graph_c0 | 2.70E-05 | -0.8467 | down | uncharacterized protein LOC111421016 [Onthophagus taurus] |
| c79574.graph_c1 | 1.31E-04 | -0.8492 | down | uncharacterized protein LOC111513514 [Leptinotarsa decemlineata] |
| c81049.graph_c1 | 2.72E-08 | -0.8555 | down | PREDICTED: selenium-binding protein 1 [Tribolium castaneum] |
| c67993.graph_c0 | 1.80E-03 | -0.8599 | down | cysteine dioxygenase type 1 [Leptinotarsa decemlineata] |
| c70776.graph_c0 | 4.72E-03 | -0.8616 | down | None |
| c76109.graph_c0 | 3.46E-05 | -0.8622 | down | glycoside hydrolase family 1 [Phyllotreta striolata] |
| c70341.graph_c0 | 5.66E-03 | -0.8633 | down | beta-glucuronidase [Anoplophora glabripennis] |
| c61767.graph_c0 | 3.10E-04 | -0.8651 | down | cuticle protein 8-like [Leptinotarsa decemlineata] |
| c74170.graph_c0 | 4.01E-03 | -0.8656 | down | PREDICTED: D-arabinitol dehydrogenase 1 [Tribolium castaneum] |
| c83031.graph_c0 | 1.71E-03 | -0.8703 | down | probable cytochrome P450 6a13 [Leptinotarsa decemlineata] |
| c68816.graph_c0 | 1.56E-03 | -0.8721 | down | odorant-binding protein [Galeruca daurica] |
| c79880.graph_c0 | 3.22E-03 | -0.8721 | down | facilitated trehalose transporter Tret1-like isoform X1 [Leptinotarsa decemlineata] |
| c83170.graph_c1 | 6.93E-03 | -0.8768 | down | PREDICTED: uncharacterized protein LOC108577227 [Habropoda laboriosa] |
| c64263.graph_c0 | 1.78E-03 | -0.8775 | down | None |
| c74442.graph_c0 | 4.75E-03 | -0.8791 | down | uncharacterized protein LOC111511653 [Leptinotarsa decemlineata] |
| c84528.graph_c0 | 3.19E-03 | -0.8817 | down | cytochrome P450 4C1-like [Anoplophora glabripennis] |
| c38861.graph_c0 | 2.61E-04 | -0.8840 | down | uncharacterized protein LOC108917609 [Anoplophora glabripennis] |
| c55212.graph_c0 | 1.62E-05 | -0.8857 | down | -- |
| c65728.graph_c0 | 5.03E-03 | -0.8864 | down | aminomethyltransferase, mitochondrial [Anoplophora glabripennis] |
| c49296.graph_c0 | 2.24E-05 | -0.8869 | down | None |
| c63934.graph_c0 | 9.18E-04 | -0.8870 | down | PREDICTED: prosaposin-like [Acropora digitifera] |
| c81494.graph_c1 | 6.22E-04 | -0.8883 | down | PREDICTED: chymotrypsin inhibitor [Trachymyrmex zeteki] |
| c66062.graph_c0 | 2.74E-04 | -0.8889 | down | endoglucanase-like [Leptinotarsa decemlineata] |
| c67871.graph_c0 | 1.09E-05 | -0.8889 | down | glycine N-methyltransferase [Anoplophora glabripennis] |
| c79283.graph_c0 | 5.69E-07 | -0.8924 | down | glycoside hydrolase family 1 [Phyllotreta striolata] |
| c54377.graph_c0 | 2.31E-06 | -0.8971 | down | glycine-rich cell wall structural protein-like isoform X2 [Anoplophora glabripennis] |
| c70204.graph_c0 | 7.13E-04 | -0.9008 | down | None |
| c66824.graph_c0 | 7.36E-04 | -0.9014 | down | None |
| c67640.graph_c0 | 1.05E-03 | -0.9033 | down | odorant-binding protein [Galeruca daurica] |
| c79225.graph_c0 | 9.30E-03 | -0.9052 | down | facilitated trehalose transporter Tret1-like [Anoplophora glabripennis] |
| c66652.graph_c1 | 4.02E-04 | -0.9121 | down | spidroin-1-like [Leptinotarsa decemlineata] |
| c37865.graph_c0 | 2.08E-04 | -0.9144 | down | PREDICTED: ovomucoid-like [Nicrophorus vespilloides] |
| c77741.graph_c0 | 1.90E-03 | -0.9177 | down | uncharacterized protein LOC111510432 [Leptinotarsa decemlineata] |
| c74169.graph_c0 | 1.63E-03 | -0.9187 | down | PREDICTED: chondroadherin-like [Aethina tumida] |
| c24767.graph_c0 | 6.04E-07 | -0.9193 | down | xyloglucan-specific endo-beta-1,4-glucanase [Gastrophysa viridula] |
| c22450.graph_c0 | 6.23E-03 | -0.9230 | down | hypothetical protein TcasGA2_TC012038 [Tribolium castaneum] |
| c75822.graph_c0 | 7.27E-04 | -0.9262 | down | protein yellow-like [Leptinotarsa decemlineata] |
| c71827.graph_c0 | 1.15E-03 | -0.9314 | down | purine nucleoside phosphorylase isoform X3 [Leptinotarsa decemlineata] |
| c83007.graph_c0 | 4.52E-05 | -0.9316 | down | uncharacterized protein LOC111503175 isoform X2 [Leptinotarsa decemlineata] |
| c80085.graph_c0 | 9.23E-03 | -0.9325 | down | PREDICTED: uncharacterized protein LOC109534924 [Dendroctonus ponderosae] |
| c58730.graph_c1 | 1.62E-03 | -0.9329 | down | peptidoglycan-recognition protein-SC2 [Tenebrio molitor] |
| c79729.graph_c0 | 9.34E-04 | -0.9330 | down | encapsulation-relating protein [Diabrotica virgifera virgifera] |
| c72057.graph_c0 | 1.08E-03 | -0.9356 | down | PREDICTED: uncharacterized protein LOC109598355 [Aethina tumida] |
| c69643.graph_c1 | 1.16E-05 | -0.9380 | down | retinoid-inducible serine carboxypeptidase-like [Leptinotarsa decemlineata] |
| c89197.graph_c0 | 3.82E-04 | -0.9405 | down | putative leucine-rich repeat-containing protein DDB_G0290503 [Leptinotarsa decemlineata] |
| c87948.graph_c0 | 6.73E-03 | -0.9468 | down | hypothetical protein RF55_9871 [Lasius niger] |
| c37977.graph_c0 | 8.65E-10 | -0.9484 | down | hypothetical protein TcasGA2_TC034814 [Tribolium castaneum] |
| c71232.graph_c0 | 2.11E-06 | -0.9496 | down | aldose 1-epimerase-like [Leptinotarsa decemlineata] |
| c70760.graph_c0 | 9.34E-04 | -0.9501 | down | None |
| c65510.graph_c0 | 2.62E-06 | -0.9506 | down | flexible cuticle protein 12-like [Leptinotarsa decemlineata] |
| c77690.graph_c0 | 7.54E-10 | -0.9541 | down | glycoside hydrolase family 1 [Phyllotreta striolata] |
| c40090.graph_c0 | 6.92E-06 | -0.9663 | down | PREDICTED: serine protease inhibitor dipetalogastin-like [Nicrophorus vespilloides] |
| c37946.graph_c0 | 6.64E-09 | -0.9667 | down | crustapain-like [Leptinotarsa decemlineata] |
| c62230.graph_c0 | 4.76E-04 | -0.9677 | down | None |
| c58730.graph_c0 | 9.92E-03 | -0.9692 | down | PREDICTED: peptidoglycan-recognition protein SC2 [Tribolium castaneum] |
| c78916.graph_c0 | 1.47E-03 | -0.9728 | down | uncharacterized protein LOC108917603 [Anoplophora glabripennis] |
| c61864.graph_c0 | 1.83E-08 | -0.9735 | down | endo-beta-1,4-glucanase [Leptinotarsa decemlineata] |
| c79778.graph_c0 | 7.67E-06 | -0.9786 | down | uncharacterized protein LOC111502712 [Leptinotarsa decemlineata] |
| c69091.graph_c0 | 7.65E-04 | -0.9791 | down | None |
| c79880.graph_c1 | 6.45E-03 | -0.9805 | down | facilitated trehalose transporter Tret1 [Anoplophora glabripennis] |
| c24797.graph_c0 | 3.67E-03 | -0.9848 | down | uncharacterized protein LOC111692798 [Anoplophora glabripennis] |
| c78986.graph_c0 | 2.31E-07 | -0.9852 | down | None |
| c58870.graph_c0 | 8.01E-03 | -0.9858 | down | uncharacterized protein LOC111515870 [Leptinotarsa decemlineata] |
| c74938.graph_c0 | 5.40E-03 | -0.9887 | down | uncharacterized protein LOC111506477 [Leptinotarsa decemlineata] |
| c86552.graph_c0 | 4.33E-03 | -0.9998 | down | titin-like [Anoplophora glabripennis] |
| c39060.graph_c0 | 8.07E-07 | -0.9999 | down | None |
| c83974.graph_c0 | 9.20E-06 | -1.0025 | down | trifunctional purine biosynthetic protein adenosine-3 [Aethina tumida] |
| c72310.graph_c0 | 1.92E-06 | -1.0037 | down | -- |
| c74532.graph_c0 | 9.98E-04 | -1.0066 | down | juvenile hormone epoxide hydrolase-like [Anoplophora glabripennis] |
| c79751.graph_c1 | 1.09E-04 | -1.0148 | down | esterase, partial [Leptinotarsa decemlineata] |
| c75113.graph_c0 | 6.97E-04 | -1.0165 | down | PREDICTED: titin isoform X3 [Tribolium castaneum] |
| c59503.graph_c0 | 1.76E-03 | -1.0170 | down | None |
| c39851.graph_c0 | 1.72E-04 | -1.0229 | down | glycoside hydrolase family 45 protein [Phaedon cochleariae] |
| c79084.graph_c3 | 4.27E-03 | -1.0259 | down | None |
| c82416.graph_c0 | 8.14E-04 | -1.0284 | down | uncharacterized protein LOC108905167 [Anoplophora glabripennis] |
| c69603.graph_c0 | 4.65E-03 | -1.0287 | down | alpha-tocopherol transfer protein-like [Anoplophora glabripennis] |
| c67343.graph_c0 | 3.69E-07 | -1.0300 | down | uncharacterized protein LOC108915662 [Anoplophora glabripennis] |
| c88018.graph_c0 | 3.92E-03 | -1.0360 | down | None |
| c88269.graph_c0 | 2.92E-07 | -1.0522 | down | uncharacterized protein LOC111692419 [Anoplophora glabripennis] |
| c76013.graph_c0 | 1.11E-03 | -1.0531 | down | mitochondrial basic amino acids transporter-like [Leptinotarsa decemlineata] |
| c87944.graph_c0 | 1.21E-03 | -1.0543 | down | transposable element tc3 transposase [Lasius niger] |
| c78814.graph_c0 | 2.60E-03 | -1.0560 | down | glycine-rich cell wall structural protein-like [Anoplophora glabripennis] |
| c75182.graph_c0 | 2.00E-03 | -1.0576 | down | uncharacterized protein LOC105841502 [Bombyx mori] |
| c68470.graph_c0 | 3.69E-04 | -1.0609 | down | None |
| c64821.graph_c0 | 3.05E-04 | -1.0611 | down | None |
| c80907.graph_c0 | 3.86E-03 | -1.0666 | down | PREDICTED: uncharacterized protein LOC101238582 [Hydra vulgaris] |
| c85849.graph_c0 | 5.86E-04 | -1.0673 | down | None |
| c74925.graph_c0 | 1.58E-04 | -1.0682 | down | myrosinase 1-like isoform X1 [Leptinotarsa decemlineata] |
| c55586.graph_c0 | 2.97E-03 | -1.0746 | down | None |
| c71366.graph_c0 | 1.43E-03 | -1.0759 | down | uncharacterized protein LOC110371565 [Helicoverpa armigera] |
| c78532.graph_c0 | 1.29E-04 | -1.0776 | down | PREDICTED: selenium-binding protein 1 [Tribolium castaneum] |
| c85079.graph_c0 | 2.14E-04 | -1.0855 | down | leucine-rich repeat-containing protein 15-like [Leptinotarsa decemlineata] |
| c81549.graph_c0 | 1.39E-03 | -1.0912 | down | leucine-rich repeat-containing G-protein coupled receptor 4-like [Leptinotarsa decemlineata] |
| c84503.graph_c0 | 2.33E-03 | -1.0912 | down | uncharacterized protein LOC111508109 [Leptinotarsa decemlineata] |
| c73627.graph_c0 | 4.34E-06 | -1.0913 | down | None |
| c79976.graph_c0 | 1.28E-04 | -1.1009 | down | dopamine N-acetyltransferase-like [Leptinotarsa decemlineata] |
| c71865.graph_c1 | 2.80E-04 | -1.1026 | down | elastin-like [Anoplophora glabripennis] |
| c88646.graph_c0 | 3.91E-03 | -1.1140 | down | None |
| c85339.graph_c0 | 7.64E-07 | -1.1146 | down | None |
| c70233.graph_c0 | 9.34E-05 | -1.1177 | down | odorant-binding protein 7, partial [Pyrrhalta aenescens] |
| c80142.graph_c0 | 1.37E-05 | -1.1194 | down | D-2-hydroxyglutarate dehydrogenase, mitochondrial-like [Leptinotarsa decemlineata] |
| c86897.graph_c0 | 8.51E-08 | -1.1225 | down | parathyroid hormone/parathyroid hormone-related peptide receptor [Anoplophora glabripennis] |
| c81902.graph_c0 | 1.36E-08 | -1.1265 | down | inter-alpha-trypsin inhibitor heavy chain H4-like isoform X2 [Leptinotarsa decemlineata] |
| c72318.graph_c0 | 2.92E-03 | -1.1284 | down | oleosin-B4-like [Leptinotarsa decemlineata] |
| c81309.graph_c1 | 1.77E-03 | -1.1314 | down | None |
| c72204.graph_c0 | 6.08E-04 | -1.1326 | down | None |
| c76386.graph_c0 | 1.86E-03 | -1.1350 | down | juvenile hormone esterase-like [Leptinotarsa decemlineata] |
| c78105.graph_c2 | 8.37E-04 | -1.1427 | down | Retrovirus-related Pol polyprotein from transposon 412, partial [Stegodyphus mimosarum] |
| c85078.graph_c0 | 8.33E-04 | -1.1435 | down | PREDICTED: LOW QUALITY PROTEIN: putative nuclease HARBI1 [Trachymyrmex zeteki] |
| c65704.graph_c0 | 4.00E-04 | -1.1480 | down | parathyroid hormone/parathyroid hormone-related peptide receptor [Anoplophora glabripennis] |
| c82858.graph_c0 | 2.58E-05 | -1.1506 | down | nephrin isoform X1 [Leptinotarsa decemlineata] |
| c61216.graph_c0 | 4.28E-07 | -1.1535 | down | uncharacterized protein LOC108903441 [Anoplophora glabripennis] |
| c74156.graph_c0 | 6.45E-06 | -1.1575 | down | UDP-glucuronosyltransferase 2B33-like [Anoplophora glabripennis] |
| c65874.graph_c0 | 1.75E-10 | -1.1588 | down | uncharacterized protein LOC111502600 [Leptinotarsa decemlineata] |
| c83806.graph_c0 | 5.86E-05 | -1.1622 | down | amidophosphoribosyltransferase-like [Leptinotarsa decemlineata] |
| c87588.graph_c0 | 2.38E-03 | -1.1650 | down | uncharacterized protein LOC111508612 [Leptinotarsa decemlineata] |
| c78450.graph_c0 | 2.63E-04 | -1.1671 | down | macrophage mannose receptor 1 [Anoplophora glabripennis] |
| c68825.graph_c0 | 2.37E-04 | -1.1738 | down | crustapain-like [Leptinotarsa decemlineata] |
| c71051.graph_c0 | 2.13E-03 | -1.1758 | down | None |
| c71233.graph_c0 | 3.89E-05 | -1.1799 | down | retinol dehydrogenase 13-like [Anoplophora glabripennis] |
| c85324.graph_c0 | 3.37E-09 | -1.1836 | down | None |
| c72150.graph_c1 | 2.72E-09 | -1.1846 | down | peptidoglycan-recognition protein 2-like [Leptinotarsa decemlineata] |
| c84542.graph_c0 | 3.66E-05 | -1.1880 | down | leucine-rich repeat-containing protein 15-like [Leptinotarsa decemlineata] |
| c50016.graph_c0 | 1.29E-05 | -1.1987 | down | sulfiredoxin-1 isoform X1 [Anoplophora glabripennis] |
| c75649.graph_c0 | 2.16E-06 | -1.1999 | down | PREDICTED: lambda-crystallin [Tribolium castaneum] |
| c68986.graph_c0 | 3.36E-04 | -1.2000 | down | cytochrome P450 4C1-like, partial [Leptinotarsa decemlineata] |
| c66705.graph_c0 | 2.24E-05 | -1.2029 | down | endocuticle structural glycoprotein SgAbd-8-like [Anoplophora glabripennis] |
| c78401.graph_c0 | 1.53E-06 | -1.2158 | down | beta-glucosidase [Nilaparvata lugens] |
| c61125.graph_c0 | 3.50E-06 | -1.2288 | down | odorant-binding protein [Galeruca daurica] |
| c82886.graph_c0 | 6.02E-04 | -1.2350 | down | nose resistant to fluoxetine protein 6-like isoform X1 [Anoplophora glabripennis] |
| c75759.graph_c0 | 1.13E-04 | -1.2458 | down | zinc carboxypeptidase [Anoplophora glabripennis] |
| c57770.graph_c0 | 5.80E-07 | -1.2471 | down | chemosensory protein [Galeruca daurica] |
| c62751.graph_c0 | 1.04E-03 | -1.2477 | down | None |
| c86956.graph_c0 | 3.97E-04 | -1.2554 | down | hypothetical protein EAG_16413 [Camponotus floridanus] |
| c81068.graph_c0 | 1.78E-12 | -1.2555 | down | venom carboxylesterase-6 isoform X1 [Anoplophora glabripennis] |
| c47940.graph_c0 | 1.42E-05 | -1.2578 | down | None |
| c81739.graph_c0 | 7.08E-04 | -1.2916 | down | integrase core domain protein [Lasius niger] |
| c69643.graph_c0 | 3.54E-06 | -1.2952 | down | retinoid-inducible serine carboxypeptidase-like [Leptinotarsa decemlineata] |
| c89552.graph_c0 | 1.23E-07 | -1.3059 | down | uncharacterized protein LOC111692544 [Anoplophora glabripennis] |
| c75023.graph_c0 | 2.30E-06 | -1.3098 | down | uncharacterized protein LOC111691687 [Anoplophora glabripennis] |
| c79635.graph_c0 | 3.79E-07 | -1.3120 | down | DNA/RNA non-specific nuclease 1 [Leptinotarsa decemlineata] |
| c73343.graph_c0 | 5.78E-04 | -1.3298 | down | None |
| c77757.graph_c0 | 9.13E-05 | -1.3342 | down | esterase FE4-like [Anoplophora glabripennis] |
| c77863.graph_c0 | 3.38E-12 | -1.3394 | down | peritrophin-1-like [Anoplophora glabripennis] |
| c55694.graph_c0 | 8.83E-06 | -1.3399 | down | inositol oxygenase [Anoplophora glabripennis] |
| c78030.graph_c0 | 1.22E-05 | -1.3445 | down | encapsulation-relating protein [Diabrotica virgifera virgifera] |
| c72307.graph_c0 | 2.95E-13 | -1.3451 | down | None |
| c81648.graph_c0 | 7.63E-07 | -1.3650 | down | regucalcin-like isoform X2 [Leptinotarsa decemlineata] |
| c69611.graph_c0 | 3.08E-04 | -1.3874 | down | None |
| c47071.graph_c0 | 1.11E-38 | -1.3895 | down | PREDICTED: crustapain-like [Dendroctonus ponderosae] |
| c38748.graph_c0 | 1.68E-08 | -1.3955 | down | None |
| c77232.graph_c0 | 4.03E-07 | -1.3959 | down | None |
| c56566.graph_c0 | 1.41E-06 | -1.4033 | down | hemolymph trypsin inhibitor B-like [Anoplophora glabripennis] |
| c79494.graph_c0 | 1.77E-04 | -1.4188 | down | fumarylacetoacetate hydrolase domain-containing protein 2-like [Leptinotarsa decemlineata] |
| c67402.graph_c0 | 3.49E-08 | -1.4346 | down | None |
| c58499.graph_c0 | 1.21E-15 | -1.5019 | down | None |
| c84589.graph_c0 | 3.12E-08 | -1.5257 | down | protein msta-like [Leptinotarsa decemlineata] |
| c75294.graph_c0 | 4.00E-08 | -1.5508 | down | arginase-1 [Anoplophora glabripennis] |
| c87806.graph_c0 | 2.94E-08 | -1.5799 | down | putative leucine-rich repeat-containing protein DDB_G0290503 [Leptinotarsa decemlineata] |
| c37561.graph_c0 | 8.49E-13 | -1.6171 | down | PREDICTED: crustapain-like [Dendroctonus ponderosae] |
| c73605.graph_c0 | 6.95E-08 | -1.6277 | down | peptidoglycan-recognition protein 2 [Anoplophora glabripennis] |
| c64462.graph_c0 | 2.70E-08 | -1.6312 | down | ras GTPase-activating protein 1-like [Anoplophora glabripennis] |
| c81637.graph_c1 | 2.16E-05 | -1.6354 | down | encapsulation-relating protein [Diabrotica virgifera virgifera] |
| c79344.graph_c0 | 5.84E-07 | -1.6565 | down | glycine-rich cell wall structural protein 1.0-like [Leptinotarsa decemlineata] |
| c81637.graph_c2 | 1.25E-05 | -1.6845 | down | encapsulation-relating protein [Diabrotica virgifera virgifera] |
| c80017.graph_c0 | 8.59E-06 | -1.6884 | down | heparan-alpha-glucosaminide N-acetyltransferase-like [Anoplophora glabripennis] |
| c82459.graph_c0 | 4.93E-06 | -1.7620 | down | None |
| c64926.graph_c0 | 2.96E-14 | -1.7772 | down | None |
| c76486.graph_c0 | 8.14E-10 | -1.8257 | down | PREDICTED: lipase member H-A-like [Dendroctonus ponderosae] |
| c71985.graph_c0 | 1.55E-10 | -1.8330 | down | attacin-B-like [Leptinotarsa decemlineata] |
| c76348.graph_c0 | 3.04E-07 | -1.9051 | down | adenylate cyclase, terminal-differentiation specific [Anoplophora glabripennis] |
| c85845.graph_c0 | 6.79E-09 | -1.9190 | down | uncharacterized protein LOC111504426 [Leptinotarsa decemlineata] |
| c68596.graph_c0 | 1.14E-12 | -2.0404 | down | RecName: Full=Tenecin-1; Flags: Precursor |
| c19745.graph_c0 | 1.01E-09 | -2.0734 | down | attacin-like immune protein [Diabrotica virgifera virgifera] |
| c72112.graph_c0 | 1.11E-33 | -2.3266 | down | facilitated trehalose transporter Tret1-like [Leptinotarsa decemlineata] |
| c38229.graph_c0 | 2.76E-15 | -2.4580 | down | putative antimicrobial peptide defensin [Phaedon cochleariae] |
| c61568.graph_c0 | 2.44E-27 | -2.5424 | down | None |
| c90260.graph_c0 | 5.78E-14 | -2.8811 | down | None |
| c74882.graph_c0 | 8.60E-23 | -3.1741 | down | uncharacterized protein LOC108905192 [Anoplophora glabripennis] |
| c78626.graph_c0 | 6.33E-125 | -4.1226 | down | bromodomain-containing protein DDB_G0280777 [Anoplophora glabripennis] |
