# Supplementary material for: Regulation of Juvenile Hormone on Summer Diapause of Geleruca daurica and Its Pathway Analysis
Source: Insects. 2021 Mar 11;12(3):237. doi: 10.3390/insects12030237 (PMC8000908; doi:10.3390/insects12030237)
Supplement: Supplementary file 1 [file insects-12-00237-s001.zip › insects-1110083-suppl-update/Table S4.docx]

| **Table S4 KEGG enrichment pathway for DEGs** | |  |  |  |  |
| --- | --- | --- | --- | --- | --- |
| **Cka vs Ta** |  |  |  |  |  |
| Pathway | ko_ID | DEG_in_Pathway | All Gene_in_Pathway | P-value | Corrected_P-value |
| Glycerophospholipid metabolism | ko00564 | 5 | 97 | 0.0080 | 0.5460 |
| Galactose metabolism | ko00052 | 3 | 37 | 0.0118 | 0.8038 |
| Lysosome | ko04142 | 6 | 151 | 0.0128 | 0.8711 |
| Arginine and proline metabolism | ko00330 | 3 | 39 | 0.0137 | 0.9283 |
| Tyrosine metabolism | ko00350 | 2 | 19 | 0.0246 | 1.0000 |
| Apoptosis - multiple species | ko04215 | 2 | 21 | 0.0298 | 1.0000 |
| Pentose and glucuronate interconversions | ko00040 | 3 | 54 | 0.0323 | 1.0000 |
| Ether lipid metabolism | ko00565 | 2 | 23 | 0.0353 | 1.0000 |
| Lipoic acid metabolism | ko00785 | 1 | 3 | 0.0385 | 1.0000 |
| Apoptosis - fly | ko04214 | 3 | 64 | 0.0496 | 1.0000 |
| Starch and sucrose metabolism | ko00500 | 3 | 66 | 0.0535 | 1.0000 |
| Longevity regulating pathway - multiple species | ko04213 | 3 | 68 | 0.0576 | 1.0000 |
| Pyrimidine metabolism | ko00240 | 5 | 165 | 0.0616 | 1.0000 |
| Purine metabolism | ko00230 | 6 | 221 | 0.0651 | 1.0000 |
| Fatty acid biosynthesis | ko00061 | 2 | 33 | 0.0678 | 1.0000 |
| Alanine, aspartate and glutamate metabolism | ko00250 | 2 | 35 | 0.0752 | 1.0000 |
| Phenylalanine, tyrosine and tryptophan biosynthesis | ko00400 | 1 | 6 | 0.0755 | 1.0000 |
| FoxO signaling pathway | ko04068 | 3 | 79 | 0.0824 | 1.0000 |
| Glycolysis / Gluconeogenesis | ko00010 | 3 | 84 | 0.0949 | 1.0000 |
| Drug metabolism - other enzymes | ko00983 | 2 | 45 | 0.1153 | 1.0000 |
| Porphyrin and chlorophyll metabolism | ko00860 | 2 | 46 | 0.1196 | 1.0000 |
| Cysteine and methionine metabolism | ko00270 | 2 | 49 | 0.1327 | 1.0000 |
| Phenylalanine metabolism | ko00360 | 1 | 11 | 0.1342 | 1.0000 |
| One carbon pool by folate | ko00670 | 1 | 12 | 0.1455 | 1.0000 |
| Apoptosis | ko04210 | 1 | 12 | 0.1455 | 1.0000 |
| Peroxisome | ko04146 | 3 | 103 | 0.1489 | 1.0000 |
| Folate biosynthesis | ko00790 | 1 | 13 | 0.1566 | 1.0000 |
| Pyruvate metabolism | ko00620 | 2 | 59 | 0.1782 | 1.0000 |
| Arginine biosynthesis | ko00220 | 1 | 15 | 0.1785 | 1.0000 |
| Glycosphingolipid biosynthesis - globo series | ko00603 | 1 | 15 | 0.1785 | 1.0000 |
| Nicotinate and nicotinamide metabolism | ko00760 | 1 | 15 | 0.1785 | 1.0000 |
| Sulfur metabolism | ko00920 | 1 | 15 | 0.1785 | 1.0000 |
| Steroid biosynthesis | ko00100 | 1 | 17 | 0.1998 | 1.0000 |
| Glycerolipid metabolism | ko00561 | 2 | 66 | 0.2114 | 1.0000 |
| Glycosaminoglycan degradation | ko00531 | 1 | 19 | 0.2205 | 1.0000 |
| Fatty acid metabolism | ko01212 | 2 | 81 | 0.2840 | 1.0000 |
| 2-Oxocarboxylic acid metabolism | ko01210 | 1 | 28 | 0.3076 | 1.0000 |
| ABC transporters | ko02010 | 2 | 88 | 0.3179 | 1.0000 |
| Retinol metabolism | ko00830 | 1 | 30 | 0.3256 | 1.0000 |
| Ascorbate and aldarate metabolism | ko00053 | 1 | 31 | 0.3344 | 1.0000 |
| Glycine, serine and threonine metabolism | ko00260 | 1 | 37 | 0.3850 | 1.0000 |
| Biosynthesis of amino acids | ko01230 | 2 | 103 | 0.3891 | 1.0000 |
| Propanoate metabolism | ko00640 | 1 | 38 | 0.3931 | 1.0000 |
| Tryptophan metabolism | ko00380 | 1 | 38 | 0.3931 | 1.0000 |
| Glyoxylate and dicarboxylate metabolism | ko00630 | 1 | 40 | 0.4089 | 1.0000 |
| Drug metabolism - cytochrome P450 | ko00982 | 1 | 40 | 0.4089 | 1.0000 |
| Sphingolipid metabolism | ko00600 | 1 | 41 | 0.4167 | 1.0000 |
| Metabolism of xenobiotics by cytochrome P450 | ko00980 | 1 | 43 | 0.4319 | 1.0000 |
| Fructose and mannose metabolism | ko00051 | 1 | 45 | 0.4467 | 1.0000 |
| Inositol phosphate metabolism | ko00562 | 1 | 55 | 0.5153 | 1.0000 |
| Citrate cycle (TCA cycle) | ko00020 | 1 | 60 | 0.5464 | 1.0000 |
| Hippo signaling pathway - fly | ko04391 | 1 | 62 | 0.5583 | 1.0000 |
| Glutathione metabolism | ko00480 | 1 | 74 | 0.6235 | 1.0000 |
| RNA polymerase | ko03020 | 1 | 74 | 0.6235 | 1.0000 |
| Phosphatidylinositol signaling system | ko04070 | 1 | 81 | 0.6570 | 1.0000 |
| Carbon metabolism | ko01200 | 2 | 172 | 0.6621 | 1.0000 |
| Endocytosis | ko04144 | 2 | 175 | 0.6715 | 1.0000 |
| Proteasome | ko03050 | 1 | 94 | 0.7117 | 1.0000 |
| RNA degradation | ko03018 | 1 | 99 | 0.7304 | 1.0000 |
| MAPK signaling pathway - fly | ko04013 | 1 | 108 | 0.7610 | 1.0000 |
| mTOR signaling pathway | ko04150 | 1 | 117 | 0.7883 | 1.0000 |
| mRNA surveillance pathway | ko03015 | 1 | 117 | 0.7883 | 1.0000 |
| Phagosome | ko04145 | 1 | 121 | 0.7994 | 1.0000 |
| Ribosome biogenesis in eukaryotes | ko03008 | 1 | 144 | 0.8529 | 1.0000 |
| RNA transport | ko03013 | 1 | 187 | 0.9181 | 1.0000 |
| Protein processing in endoplasmic reticulum | ko04141 | 1 | 199 | 0.9305 | 1.0000 |
| Spliceosome | ko03040 | 1 | 218 | 0.9465 | 1.0000 |
| Ribosome | ko03010 | 1 | 259 | 0.9697 | 1.0000 |
| **Ckb vs Tb** |  |  |  |  |  |
| Pathway | ko_ID | DEG_in_Pathway | AllGene_in_Pathway | P-value | Corrected_P-value |
| Drug metabolism - cytochrome P450 | ko00982 | 6 | 40 | 0.0005 | 0.0454 |
| Metabolism of xenobiotics by cytochrome P450 | ko00980 | 6 | 43 | 0.0008 | 0.0677 |
| Taurine and hypotaurine metabolism | ko00430 | 3 | 11 | 0.0025 | 0.2139 |
| Pentose and glucuronate interconversions | ko00040 | 6 | 54 | 0.0026 | 0.2269 |
| Glutathione metabolism | ko00480 | 7 | 74 | 0.0030 | 0.2572 |
| One carbon pool by folate | ko00670 | 3 | 12 | 0.0033 | 0.2798 |
| Lysosome | ko04142 | 10 | 151 | 0.0058 | 0.4993 |
| Drug metabolism - other enzymes | ko00983 | 5 | 45 | 0.0060 | 0.5171 |
| Ascorbate and aldarate metabolism | ko00053 | 4 | 31 | 0.0082 | 0.7032 |
| Glycosaminoglycan degradation | ko00531 | 3 | 19 | 0.0125 | 1.0000 |
| Herpes simplex infection | ko05168 | 2 | 8 | 0.0172 | 1.0000 |
| Other glycan degradation | ko00511 | 3 | 23 | 0.0212 | 1.0000 |
| Insect hormone biosynthesis | ko00981 | 3 | 25 | 0.0266 | 1.0000 |
| Starch and sucrose metabolism | ko00500 | 5 | 66 | 0.0285 | 1.0000 |
| Porphyrin and chlorophyll metabolism | ko00860 | 4 | 46 | 0.0314 | 1.0000 |
| Cysteine and methionine metabolism | ko00270 | 4 | 49 | 0.0385 | 1.0000 |
| Histidine metabolism | ko00340 | 2 | 13 | 0.0441 | 1.0000 |
| Basal transcription factors | ko03022 | 5 | 75 | 0.0459 | 1.0000 |
| Fatty acid biosynthesis | ko00061 | 3 | 33 | 0.0542 | 1.0000 |
| Galactose metabolism | ko00052 | 3 | 37 | 0.0717 | 1.0000 |
| ABC transporters | ko02010 | 5 | 88 | 0.0800 | 1.0000 |
| mRNA surveillance pathway | ko03015 | 6 | 117 | 0.0855 | 1.0000 |
| DNA replication | ko03030 | 5 | 94 | 0.0994 | 1.0000 |
| Progesterone-mediated oocyte maturation | ko04914 | 1 | 6 | 0.1475 | 1.0000 |
| Glycolysis / Gluconeogenesis | ko00010 | 4 | 84 | 0.1776 | 1.0000 |
| Retinol metabolism | ko00830 | 2 | 30 | 0.1852 | 1.0000 |
| Mismatch repair | ko03430 | 3 | 61 | 0.2145 | 1.0000 |
| Pentose phosphate pathway | ko00030 | 2 | 35 | 0.2336 | 1.0000 |
| Alanine, aspartate and glutamate metabolism | ko00250 | 2 | 35 | 0.2336 | 1.0000 |
| Nucleotide excision repair | ko03420 | 4 | 97 | 0.2495 | 1.0000 |
| Glycerophospholipid metabolism | ko00564 | 4 | 97 | 0.2495 | 1.0000 |
| Glycine, serine and threonine metabolism | ko00260 | 2 | 37 | 0.2532 | 1.0000 |
| Phenylalanine metabolism | ko00360 | 1 | 11 | 0.2538 | 1.0000 |
| Oocyte meiosis | ko04114 | 1 | 11 | 0.2538 | 1.0000 |
| Tryptophan metabolism | ko00380 | 2 | 38 | 0.2630 | 1.0000 |
| Arginine and proline metabolism | ko00330 | 2 | 39 | 0.2728 | 1.0000 |
| Apoptosis | ko04210 | 1 | 12 | 0.2734 | 1.0000 |
| Folate biosynthesis | ko00790 | 1 | 13 | 0.2926 | 1.0000 |
| RNA polymerase | ko03020 | 3 | 74 | 0.3070 | 1.0000 |
| Nicotinate and nicotinamide metabolism | ko00760 | 1 | 15 | 0.3293 | 1.0000 |
| Sulfur metabolism | ko00920 | 1 | 15 | 0.3293 | 1.0000 |
| Glycosphingolipid biosynthesis - globo series | ko00603 | 1 | 15 | 0.3293 | 1.0000 |
| Cell cycle | ko04110 | 1 | 15 | 0.3293 | 1.0000 |
| Arginine biosynthesis | ko00220 | 1 | 15 | 0.3293 | 1.0000 |
| Fructose and mannose metabolism | ko00051 | 2 | 45 | 0.3315 | 1.0000 |
| FoxO signaling pathway | ko04068 | 3 | 79 | 0.3433 | 1.0000 |
| Selenocompound metabolism | ko00450 | 1 | 16 | 0.3470 | 1.0000 |
| Fatty acid metabolism | ko01212 | 3 | 81 | 0.3578 | 1.0000 |
| Purine metabolism | ko00230 | 7 | 221 | 0.3598 | 1.0000 |
| Other types of O-glycan biosynthesis | ko00514 | 1 | 18 | 0.3809 | 1.0000 |
| Tyrosine metabolism | ko00350 | 1 | 19 | 0.3972 | 1.0000 |
| Inositol phosphate metabolism | ko00562 | 2 | 55 | 0.4258 | 1.0000 |
| Fatty acid elongation | ko00062 | 1 | 21 | 0.4286 | 1.0000 |
| Apoptosis - multiple species | ko04215 | 1 | 21 | 0.4286 | 1.0000 |
| Pyrimidine metabolism | ko00240 | 5 | 165 | 0.4371 | 1.0000 |
| Fanconi anemia pathway | ko03460 | 2 | 58 | 0.4528 | 1.0000 |
| Ether lipid metabolism | ko00565 | 1 | 23 | 0.4583 | 1.0000 |
| Arachidonic acid metabolism | ko00590 | 1 | 23 | 0.4583 | 1.0000 |
| Valine, leucine and isoleucine degradation | ko00280 | 2 | 61 | 0.4791 | 1.0000 |
| Biosynthesis of amino acids | ko01230 | 3 | 103 | 0.5113 | 1.0000 |
| Glycerolipid metabolism | ko00561 | 2 | 66 | 0.5212 | 1.0000 |
| Ribosome biogenesis in eukaryotes | ko03008 | 4 | 144 | 0.5268 | 1.0000 |
| RNA transport | ko03013 | 5 | 187 | 0.5486 | 1.0000 |
| beta-Alanine metabolism | ko00410 | 1 | 30 | 0.5508 | 1.0000 |
| Neuroactive ligand-receptor interaction | ko04080 | 2 | 71 | 0.5609 | 1.0000 |
| Ubiquitin mediated proteolysis | ko04120 | 4 | 160 | 0.6113 | 1.0000 |
| Amino sugar and nucleotide sugar metabolism | ko00520 | 2 | 83 | 0.6466 | 1.0000 |
| Sphingolipid metabolism | ko00600 | 1 | 41 | 0.6655 | 1.0000 |
| Aminoacyl-tRNA biosynthesis | ko00970 | 2 | 86 | 0.6659 | 1.0000 |
| TGF-beta signaling pathway | ko04350 | 1 | 42 | 0.6744 | 1.0000 |
| N-Glycan biosynthesis | ko00510 | 1 | 42 | 0.6744 | 1.0000 |
| Proteasome | ko03050 | 2 | 94 | 0.7132 | 1.0000 |
| Fatty acid degradation | ko00071 | 1 | 49 | 0.7302 | 1.0000 |
| Protein processing in endoplasmic reticulum | ko04141 | 4 | 199 | 0.7752 | 1.0000 |
| Pyruvate metabolism | ko00620 | 1 | 59 | 0.7939 | 1.0000 |
| Apoptosis - fly | ko04214 | 1 | 64 | 0.8199 | 1.0000 |
| Phagosome | ko04145 | 2 | 121 | 0.8334 | 1.0000 |
| Longevity regulating pathway - multiple species | ko04213 | 1 | 68 | 0.8384 | 1.0000 |
| Lysine degradation | ko00310 | 1 | 71 | 0.8510 | 1.0000 |
| Phosphatidylinositol signaling system | ko04070 | 1 | 81 | 0.8863 | 1.0000 |
| Peroxisome | ko04146 | 1 | 103 | 0.9375 | 1.0000 |
| Carbon metabolism | ko01200 | 2 | 172 | 0.9453 | 1.0000 |
| MAPK signaling pathway - fly | ko04013 | 1 | 108 | 0.9455 | 1.0000 |
| Endocytosis | ko04144 | 2 | 175 | 0.9489 | 1.0000 |
| Ribosome | ko03010 | 2 | 259 | 0.9931 | 1.0000 |
| Spliceosome | ko03040 | 1 | 218 | 0.9974 | 1.0000 |
