# Supplementary material for: Regulation of Juvenile Hormone on Summer Diapause of Geleruca daurica and Its Pathway Analysis
Source: Insects. 2021 Mar 11;12(3):237. doi: 10.3390/insects12030237 (PMC8000908; doi:10.3390/insects12030237)
Supplement: Supplementary file 1 [file insects-12-00237-s001.zip › insects-1110083-suppl-update/Table S5 .docx]

Table S5 Significantly enriched GO terms for the DEGs

| **GO.ID** | **Term** | **DEG number** | **Corrected-*p_*value** |
| --- | --- | --- | --- |
| **CKa vs Ta** |  |  |  |
| **Biological process** | |  |  |
| GO:0055114 | oxidation-reduction process | 1 | 0.0023 |
| GO:0090304 | nucleic acid metabolic process | 1 | 0.0027 |
| GO:0032502 | developmental process | 1 | 0.0080 |
| GO:0044703 | multi-organism reproductive process | 1 | 0.0085 |
| GO:0032501 | multicellular organismal process | 1 | 0.0096 |
| GO:0051705 | multi-organism behavior | 1 | 0.0104 |
| GO:0019098 | reproductive behavior | 1 | 0.0105 |
| GO:0044705 | multi-organism reproductive behavior | 1 | 0.0105 |
| GO:0044767 | single-organism developmental process | 1 | 0.0113 |
| GO:0033057 | multicellular organismal reproductive behavior | 1 | 0.0141 |
| GO:0044706 | multi-multicellular organism process | 1 | 0.0141 |
| GO:0000003 | reproduction | 1 | 0.0143 |
| GO:0044707 | single-multicellular organism process | 1 | 0.0153 |
| GO:0048609 | multicellular organismal reproductive process | 1 | 0.0195 |
| GO:0019953 | sexual reproduction | 1 | 0.0212 |
| GO:0007275 | multicellular organismal development | 1 | 0.0214 |
| GO:0007610 | behavior | 1 | 0.0254 |
| GO:0048856 | anatomical structure development | 1 | 0.0462 |
| GO:0007617 | mating behavior | 1 | 0.0479 |
| GO:0007618 | mating | 1 | 0.0479 |
| **Cellular component** | |  |  |
| GO:0030529 | ribonucleoprotein complex | 1 | 0.0086 |
| GO:0005737 | cytoplasm | 2 | 0.0115 |
| GO:0071944 | cell periphery | 1 | 0.0278 |
| GO:0000323 | lytic vacuole | 1 | 0.0314 |
| GO:0005764 | lysosome | 1 | 0.0314 |
| GO:0044424 | intracellular part | 3 | 0.0331 |
| GO:0005773 | vacuole | 1 | 0.0369 |
| GO:0043227 | membrane-bounded organelle | 1 | 0.0376 |
| GO:0044444 | cytoplasmic part | 2 | 0.0393 |
| **Molecular function** | |  |  |
| GO:0003676 | nucleic acid binding | 2 | 0.0000 |
| GO:0016491 | oxidoreductase activity | 2 | 0.0009 |
| GO:0003824 | catalytic activity | 9 | 0.0226 |
| **CKb vs Tb** |  |  |  |
| **Biological process** | |  |  |
| GO:0006412 | translation | 1 | 0.0000 |
| GO:0042255 | ribosome assembly | 1 | 0.0019 |
| GO:0055114 | oxidation-reduction process | 4 | 0.0024 |
| GO:0090304 | nucleic acid metabolic process | 2 | 0.0030 |
| GO:0030163 | protein catabolic process | 1 | 0.0066 |
| GO:0042254 | ribosome biogenesis | 2 | 0.0123 |
| GO:0034660 | ncRNA metabolic process | 1 | 0.0135 |
| GO:0006520 | cellular amino acid metabolic process | 1 | 0.0147 |
| GO:0006364 | rRNA processing | 1 | 0.0166 |
| GO:0009123 | nucleoside monophosphate metabolic process | 2 | 0.0167 |
| GO:0051603 | proteolysis involved in cellular protein catabolic process | 1 | 0.0179 |
| GO:0044257 | cellular protein catabolic process | 1 | 0.0179 |
| GO:0016072 | rRNA metabolic process | 1 | 0.0197 |
| GO:0043632 | modification-dependent macromolecule catabolic process | 1 | 0.0201 |
| GO:0009161 | ribonucleoside monophosphate metabolic process | 2 | 0.0226 |
| GO:0006511 | ubiquitin-dependent protein catabolic process | 1 | 0.0227 |
| GO:1902626 | assembly of large subunit precursor of preribosome | 1 | 0.0233 |
| GO:0009167 | purine ribonucleoside monophosphate metabolic process | 2 | 0.0246 |
| GO:0009126 | purine nucleoside monophosphate metabolic process | 2 | 0.0246 |
| GO:0042274 | ribosomal small subunit biogenesis | 1 | 0.0263 |
| GO:0043436 | oxoacid metabolic process | 2 | 0.0273 |
| GO:0006082 | organic acid metabolic process | 2 | 0.0273 |
| GO:0022613 | ribonucleoprotein complex biogenesis | 2 | 0.0276 |
| GO:0019752 | carboxylic acid metabolic process | 2 | 0.0325 |
| GO:0019941 | modification-dependent protein catabolic process | 1 | 0.0332 |
| GO:0002376 | immune system process | 3 | 0.0352 |
| GO:0009124 | nucleoside monophosphate biosynthetic process | 2 | 0.0386 |
| GO:0051656 | establishment of organelle localization | 1 | 0.0416 |
| GO:0045333 | cellular respiration | 1 | 0.0427 |
| GO:0010467 | gene expression | 1 | 0.0461 |
| GO:0000028 | ribosomal small subunit assembly | 1 | 0.0464 |
| GO:0006091 | generation of precursor metabolites and energy | 1 | 0.0475 |
| **Cellular component** | |  |  |
| GO:0005840 | ribosome | 1 | 0.0000 |
| GO:0070013 | intracellular organelle lumen | 1 | 0.0024 |
| GO:0044391 | ribosomal subunit | 1 | 0.0092 |
| GO:0015935 | small ribosomal subunit | 1 | 0.0098 |
| GO:0044428 | nuclear part | 1 | 0.0108 |
| GO:0005737 | cytoplasm | 6 | 0.0119 |
| GO:0044424 | intracellular part | 6 | 0.0133 |
| GO:0030529 | ribonucleoprotein complex | 2 | 0.0145 |
| GO:0031981 | nuclear lumen | 1 | 0.0204 |
| GO:0005730 | nucleolus | 1 | 0.0286 |
| GO:0043227 | membrane-bounded organelle | 1 | 0.0434 |
| GO:0031974 | membrane-enclosed lumen | 1 | 0.0464 |
| **Molecular function** | |  |  |
| GO:0003676 | nucleic acid binding | 1 | 0.0000 |
| GO:0016491 | oxidoreductase activity | 5 | 0.0006 |
| GO:0042302 | structural constituent of cuticle | 1 | 0.0013 |
| GO:0004930 | G-protein coupled receptor activity | 1 | 0.0021 |
| GO:0003824 | catalytic activity | 20 | 0.0242 |
| GO:0016667 | oxidoreductase activity, acting on a sulfur group of donors | 1 | 0.0347 |
