# Supplementary figures and images for: Regulation of Juvenile Hormone on Summer Diapause of Geleruca daurica and Its Pathway Analysis
Source: Insects. 2021 Mar 11;12(3):237. doi: 10.3390/insects12030237 (PMC8000908; doi:10.3390/insects12030237)

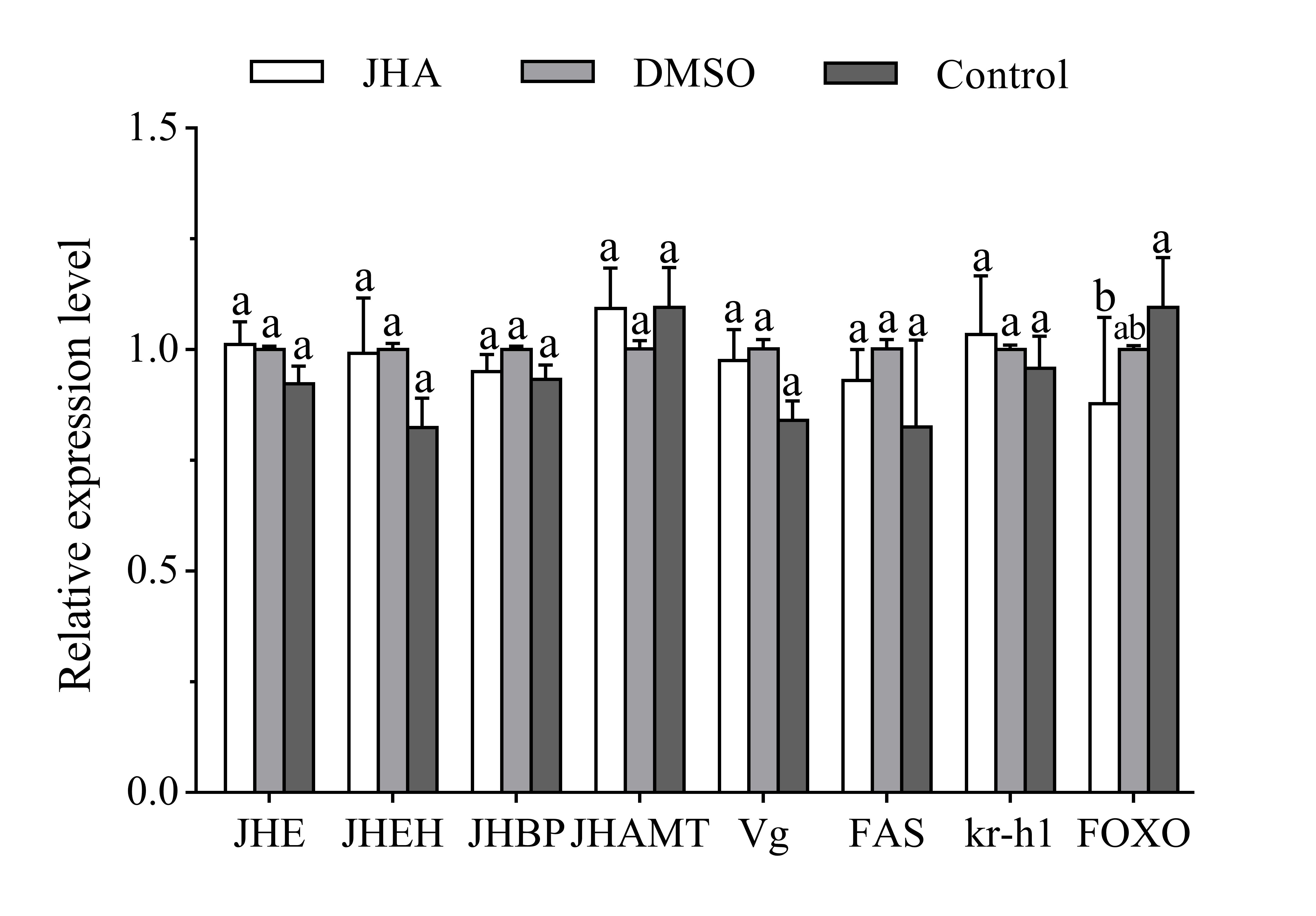

Supplement: Supplementary file 1 [file insects-12-00237-s001.zip › insects-1110083-suppl-update/Fig. S1.jpg]
